# Computational Prediction of Drug Responses in Cancer Cell Lines From Cancer Omics and Detection of Drug Effectiveness Related Methylation Sites

**DOI:** 10.3389/fgene.2020.00917

**Published:** 2020-08-07

**Authors:** Rui Yuan, Shilong Chen, Yongcui Wang

**Affiliations:** ^1^Key Laboratory of Plateau Biological Adaptation and Evolution, Northwest Institute of Plateau Biology, Chinese Academy of Sciences, Xining, China; ^2^University of Chinese Academy of Sciences, Beijing, China; ^3^Institute of Sanjiangyuan National Park, Chinese Academy of Sciences, Xining, China; ^4^Qinghai Provincial Key Laboratory of Crop Molecular Breeding, Northwest Institute of Plateau Biology, Chinese Academy of Sciences, Xining, China

**Keywords:** cancer omics, DNA methylation, drug response, GDSC database, drug effectiveness related methylation sites

## Abstract

Accurately predicting the response of a cancer patient to a therapeutic agent remains an important challenge in precision medicine. With the rise of data science, researchers have applied computational models to study the drug inhibition effects on cancers based on cancer genomics and transcriptomics. Moreover, a common epigenetic modification, DNA methylation, has been related to the occurrence and development of cancer, as well as drug effectiveness. Therefore, it is helpful for improvement of drug response prediction through exploring the relationship between DNA methylation and drug effectiveness. Here, we proposed a computational model to predict drug responses in cancers through integration of cancer genomics, transcriptomics, epigenomics, and compound chemical properties. Meanwhile, we applied a regularized regression model (Least Absolute Shrinkage and Selection Operator, lasso) to detect the methylation sites that were closely related to drug effectiveness. The prediction models were trained on a well-known pharmacogenomics data resource, Genomics of Drug Sensitivity in Cancer (GDSC). The cross-validation indicates that the performance of the prediction model using DNA methylation is comparable to that of using other cancer omics, including oncogene mutation and gene expression data. It indicates the important role of DNA methylation in prediction of drug responses. Encyclopedia of DNA Elements (ENCODE) and Transcriptional Regulatory Relationships Unraveled by Sentence-based Text mining (TRRUST2) database analyses suggest that the methylation sites associated with drug effectiveness are mainly located in the transcription factor (TF) binding region. Therefore, we hypothesized that the sensitivity of cancer cells to drugs could be regulated by changing the methylation modification of TF binding region. In conclusion, we confirmed the important role of DNA methylation in prediction of drug responses, and provided some methylation sites that closely related to the drug effectiveness, which may be a great regulatory target for improvement of drug treatment effects on cancer patients.

## Introduction

Precision medicine is a medical concept based on personalized medicine, which develops with the rapid progress of genome sequencing technology and the cross-application of biological information and big data science (Hodson, 2016). It is the ultimate goal of personalized therapy to systematically transform cancer omics into oncobiology knowledge and treatment (Barretina et al., 2012; Stetson et al., 2014; Dong et al., 2015). Accurately predicting the sensitivity of cancer cells to drug treatment is a key challenge in precision medicine.

In previous work, researchers have made significant contributions to improve drug response prediction by using large-scale pharmacogenomics data. All these works could be basically divided into two types. One of these is learning the drug responses based only on cancer omics. For instance, Costello et al. (2014) applied the multiple kernel learning algorithm to improve drug response prediction from genomic, proteomic, and epigenomic profiling data in breast cancer cell lines; Huang C. et al. (2018), Huang H.H. et al. (2018) established drug response prediction model based on the gene expression profile (RNA-seq or microarray) data of patients’ tumors. Venkatesan et al. (2010) developed a scalable and extensible prediction model by integrating genome-scale mRNA expression, copy number change, and mutation profiles. The other type is to predict the drug responses by integrating both cancer omics and chemical properties. For instance, a systematic method was proposed to determine the chemotherapy responses of cancer cell lines by integration of cancer omics and the chemical and therapeutic characteristics of compounds (Menden et al., 2013; Wang et al., 2016) integrated genomic features of cell lines (mutation, copy number, and microsatellite instability) and chemical properties of drugs to represent each cell line–drug pair and applied a neural network to predict drug responses; Zhang et al. (2015) constructed a dual-layer network model for predicting drug reactions using the proximal information of the drug and cancer cell line networks. Both two types of works have introduced the machine learning algorithms to implement the learning task, including logistic regression (LR) (Geeleher et al., 2014; Huang et al., 2020), random forest (RF) (Gregory et al., 2011; Raziur et al., 2017), support vector machine (SVM) (Burbidge et al., 2001; Ben-Hur, 2008; Wang et al., 2016), and deep learning (DL) (LeCun et al., 2015; Yoosup et al., 2018; Chiu et al., 2019).

As research deepens, epigenetic modifications have been found to be directly or indirectly linked to cancer (Jones and Baylin, 2002; Kanwal and Gupta, 2012; Mohammad et al., 2019; Zhao and Shilatifard, 2019). DNA methylation is one of the most common epigenetic modifications. Under the premise of unchanged DNA sequences, methylation occurs on the cytosine bases of CpG sequence, which will affect the transcription of downstream genes (Jones, 2012; Jones et al., 2015; Edwards et al., 2017; Andrews et al., 2018). As one of the major epigenetic modifications in biological processes or diseases, DNA methylation has been well studied in many aspects, such as functions and regulatory mechanism (Bird, 1986; Moore et al., 2013), disease or phenotype (Robertson, 2005; Baccarelli et al., 2010; Zelin and Yun, 2018), evolutionary analysis (Zemach et al., 2010), X-chromosome inactivation (Singer-Sam and Riggs, 1993), DNA methylation-related cell differentiation (Mirang and Joseph, 2017), and drug inhibition effects on cancer patients (Priebsch et al., 2006; Ye et al., 2018; Lai et al., 2019).

The emergence of high-throughput drug screening technology enables us to test hundreds of drugs at the same time. The curated databases deposit the responses of thousands of cancer cells to hundreds of anti-cancer drugs, such as Genomics of Drug Sensitivity in Cancer (GDSC) and Cancer Cell Line Encyclopedia (CCLE). The GDSC project provides a large-scale collection of cancer genomic data for therapeutic biomarker discovery (Yang et al., 2013). It includes mutations for 19,100 genes across 1,001 cancer cell lines, DNA copy number variations for 46,221 genes across 996 cancer cell lines, DNA methylation (β-value) for 14,725 CpG islands across 1,029 cancer cell lines, and expression for 17,737 mRNAs across 1,018 cancer cell lines (Yang et al., 2013; Iorio et al., 2016). The CCLE project, which aims to accurately characterize the genetic characteristics of cancer cell lines, includes mutation status for 25 oncogenes across 486 cancer cell lines, DNA copy number variations for 23,316 genes across 1,043 cancer cell lines, and mRNA expressions for 54,675 mRNAs across 127 cancer cell lines (Barretina et al., 2012). In 2019, the CCLE database received a major update, including newly released DNA methylation data, whole genome sequencing data, and RNA-seq data (Ghandi et al., 2019).

Inspired by above observations, here, we assessed the contribution of DNA methylation in prediction of drug responses by comparing with that of other cancer omics via three machine learning algorithms and identified the methylation sites that were closely related to drug effectiveness through a Least Absolute Shrinkage and Selection Operator (lasso) regression model, which performs both variable selection and regularization to improve the prediction accuracy and enhance the interpretability of the statistical model (Fadil and William, 1986; Tibshirani, 1996; Yvan et al., 2007; Lockhart et al., 2014). Specifically, to integrate the heterogeneous cancer omics and compound chemical properties, the kernel-based similarity matrices were constructed to represent cancer cell lines and anti-cancer drugs, respectively. To simultaneously consider all available drugs across all cancer cell lines, a bipartite graph was introduced based on experimental drug screening results, to represent the relationships between cancer cell lines and small molecules. Here, instead of estimating the continuous response value, we categorized the response value into three classes – sensitive, resistant, and unclear – and introduced a “categorical” classifier to detect whether a given cancer cell was sensitive or resistant to a specific anti-cancer therapy. Three machine learning algorithms (LR, RF, and SVM) were introduced to train a binary classification model based on the concatenation of cancer cell and drug similarity matrix. It is worth mentioning that the data scale of this study is not suitable for DL, which depends on large-scale data size to learning the huge number of model parameters. Thus, we will not introduce the DL in this article. After testing our models on the GDSC dataset, the importance of DNA methylation in drug response prediction was suggested. Then we applied DNA methylation data to the CCLE database as an independent dataset to further assess the contribution of DNA methylation in drug response prediction. Furthermore, to detect the drug effectiveness related methylation sites, the methylation level of CpG islands were related to drug response value by lasso regression model. Encyclopedia of DNA Elements (ENCODE) and Transcriptional Regulatory Relationships Unraveled by Sentence-based Text mining (TRRUST2) database analyses suggest that the methylation sites associated with drug effectiveness are mainly located in the transcription factor (TF) binding region.

## Data Resources and Methods

### Cancer Cell Similarity

Here, we used a similar matrix to replace the original data, the purpose of which was to keep the scale of the feature and that of sample same, thereby attempting to overcome overfitting. The DNA methylation, Mutation, DNA copy number, and mRNA expression were introduced to construct the cancer cell similarity matrix.

### Mutation

GDSC provides 19,100 gene mutations in 1,001 cancer cell lines. By converting both files into a gene-by-sample matrix of binary values (1-mutation and 0-wild type), a similarity matrix was generated:

$$S_{M\,u\,t}\left(c_{i},c_{j}\right)=\exp(-\left(HD\left(c_{i},c_{j}\right)\right),$$

where *c*_i_, *c*_j_ are the binary mutation profile of the i-th and j-th cancer cell lines, respectively, and *HD*(*c_i_,c_j_*) is the Hamming distance between binary profile *c*_i_ and *c*_j_. The download link for the mutation is https://www.cancerrxgene.org/gdsc1000/GDSC1000_WebResources//Data/suppData/TableS2C.xlsx.

### DNA Copy Number

We downloaded the “cnv_20191101” zip file from GDSC. This document offered copy numbers for 24,502 gene across 986 cancer cells. We defined the cell similarity matrix based on copy number as follows:

$$S_{c\,o\,p\,y}\,\left(c_{i},c_{j}\right)=\exp\left(-\alpha \,{||c_{i}-c_{j}||}^{2}\right),$$

where *c*_i_, *c*_j_ are the copy number profile of the i-th and j-th cancer cell lines, respectively, and α is a pre-defined parameter (set as 0.001 here). The download link for the copy number is https://cog.sanger.ac.uk/cmp/download/cnv_20191101.zip.

### mRNA Expression

GDSC provides expressions for 37,279 gene across a total of 1,047 cell lines. Through the equation *S*_GE_(*c*_i_,*c*_j_) = *exp*⁡(−α||*c*_i_−*c*_j_||^2^), where *c*_i_, *c*_j_ are the expression profile of the i-th and j-th cancer cell lines, respectively, and α is a pre-defined parameter (set as 0.0001 here), the similarity between *c*_i_ and *c*_j_ was calculated. The download link for the mRNA expression is https://cog.sanger.ac.uk/cmp/download/rnaseq_20191101.zip.

### DNA Methylation

We downloaded the “METH_CELL_DATA.txt” zip file from GDSC and the “CCLE_RRBS_cgi_CpG_clusters_20181119” txt file from CCLE. The former includes β values of 14,726 islands in across 1,029 cancer cells. The latter contains 81,038 CpG islands from 843 cancer cell lines. Subsequently, we constructed the similarity matrix *S_Met__h__y_* based on these DNA methylation data: *S*_Methy_(*c*_i_,*c*_j_) = *exp*⁡(−α||*c*_i_−*c*_j_||^2^), where *c*_i_, *c*_j_ are the expression profile of the i-th and j-th cancer cell lines, respectively, and α is a pre-defined parameter (set as 0.0001 here). The download links for the DNA methylation are https://www. cancerrxgene.org/gdsc1000/GDSC1000_WebResources//Data/pr eprocessed/methylation/METH_CELL_DATA.txt.zip and https://data.broadinstitute.org/ccle/CCLE_RRBS_cgi_CpG_clusters_20181119.txt.gz.

### Drug Similarity

GDSC provides a total of 265 anti-cancer drug sensitivity data. Using QuaSAR-Descriptor in the Molecular Operating Environment (MOE v. 2011.10), we calculated the compound chemical properties for each anti-cancer drug. Specifically, the MOE descriptor created 35 features for 209 compounds, which included 2D descriptors and 3D descriptors. The chemical similarities among drugs were calculated as follows:

$$S\,i\,m_{d\,r\,u\,g\,\left(d,d^{\prime }\right)}=\exp\left(-\alpha \,{||d-d^{\prime }||}^{2}\right)$$

where *d*, *d*′ are the MOE descriptors of drug *d* and *d*′, respectively, and α is a pre-defined parameter (set as 0.001 here). Meanwhile, we applied the same method to construct the drug similarity matrix for CCLE 24 drugs. The download links for the drug responses are https://www.cancerrxgene.org/gdsc1000/GDSC1000_WebResources/Data/suppData/TableS4B.xlsx and https://data.broadinstitute.org/ccle_legacy_data/pharmacological_profiling/CCLE_NP24.2009_Drug_data_2015.02.24.csv.

### Classification Model

Three classical classification models (SVM, RF, and LR) were introduced to build the “categorical” classifier.

**FIGURE 1:**
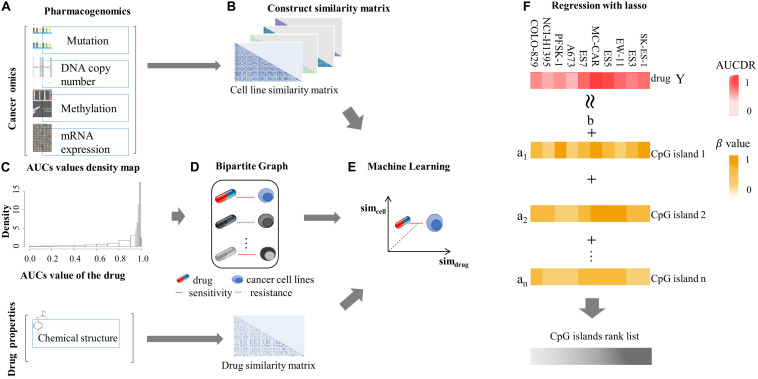
Machine learning flowchart. **(A)** Pharmacogenomic data, including mutation, DNA copy number, mRNA expression, DNA methylation, and drug sensitive data, were introduced. These data were from GDSC database. **(B)** The cell similarity and drug similarity were calculated using cancer omics and drug chemical structure, respectively. **(C)** The histogram showing the distribution of experimentally tested drug screening data: area under the dose-response curve (AUCDR). The distribution of AUCDR indicates three classes of relationships between cells and drugs, sensitive, resistant, and unclear. **(D)** Representing the known relationship between cells and drugs as a standard in the bipartite graph. **(E)** Using SVM, RF, and LR algorithms to predict the relationship between novel cells and drugs. **(F)** Using the Lasso model to predict methylation sites related to drug sensitivity.

The similarity matrix of cancer cell lines was constructed based on multiple cancer omics data sources (Figure 1A), and the similarity matrix of drugs was constructed based on the chemical properties of small molecules (Figure 1B). The input vector *X* for SVM training was defined by the concatenation of cancer cell and drug similarity matrix, that is, X= [*Sim_cell,_ Sim_drug_*] (*Sim*_cell_ could be one of*S_Mut_, S_GE_*,*S_copy_, S_Met__hy_*). The dimension of inputs is 990, 944, 943, and 897 for mutation, expression, copy number, and methylation data, respectively. We used a vector space integration (VSI) where each row of the cancer cell lines similarity matrix (*Sim*_cell_) was concatenated with corresponding row of the anti-cancer drugs similarity matrix (*Sim*_drug_). VSI is suitable for data integration independently from the structure of the involved dataset and has the advantage of simplicity (Noble and Ben-Hur, 2007). The output *Y* for classification model was a binary vector, that was obtained based on the distribution of drug screening experimental results. In our experiment, the area under the dose–response curve (AUCDR∈[0,1]) in GDSC was used to quantify the drug response in cell lines. Figure 1C shows the distribution of AUCDR of 209 drugs in 990 cell lines. According to the AUCDR distribution, we divided the response values into three categories: sensitivity, resistance, and unclear. In order to keep training positive and negative in a same scale, we defined sensitivity with AUCDR less than 0.2, and resistance with AUCDR larger than 0.991. As a result, 4,491 resistant and 3,376 sensitive pairs of cancer cell lines and drugs were achieved. In this article, we only focused on extreme cases, that is, we will not consider the cell-drug pairs classified as unclear. In particular, we constructed a bipartite map of cancer cells with known drug reactions. The nodes in these two bipartite graphs represent drugs and cell lines, respectively. The edges between cells and drugs represent their relationship, defined either as sensitivity or resistance (Figure 1D). The relationship between the cell line and the drug was represented by a bipartite graph, which was to transform the learning problem from a general binary classification task to an interaction prediction task. Its goal was to learn the drug response on a large scale, that is, learn the cancer sensitivity across lots of drugs simultaneously, in one model. The three classification models (SVM, RF, and LR), were implemented based on above input and output vectors (Figure 1E). Specifically, SVM, which is motivated by statistical learning theory (Cortes and Vapnik, 1995; Vapnik, 1999; Evgeniou et al., 2000; Vaidya et al., 2008), was implemented via “e107” R package, and the parameters were optimized by a grid search (cost = 10, RBF kernel parameter gamma = 0.01); an integrated algorithm composed of decision trees, the RF classification model (Breiman, 2001; Goldstein et al., 2011), was implemented through R “randomForest” package with default parameters; LR model, which is used to express the possibility of something happening (Liang et al., 2013; Zhao and Tang, 2018), was implemented by the R “glmnet” package with default parameters.

To evaluate the performance of the classification algorithms, the fivefold cross-validation was performed. That is, each dataset was randomly divided into five parts. Four parts were selected as the training set each time, and the remaining 1 part was applied as the test set. After five rounds, the area under the Precision Recall (PR) curve (AUPR) (Saito and Rehmsmeier, 2017) was applied to evaluate the performance of above three classification models.

### Prediction of Methylation Sites Related to Drug Responses Based on Lasso Model

To predict the methylation sites that were closely related to drug responses, the lasso regression model was introduced. The input and output for lasso were the GpG island β values across cancer cell lines and a given drug response in these cancer cell lines, respectively. Lasso regression achieved a more refined model by constructing a regularized term that compresses the regression coefficients:

$$\text{Min}\,\left(\sum_{i=1}^{m}{\left(y_{i}-W^{T}\,X\right)}^{2}+\lambda \,{||\text{W}||}_{1}\right)$$

where *y*_i_ is the AUCDR value of i-th drug, *X* is the β value of methylation sites across cancer cell lines, λ is the regularization parameter, and ||W|| _1_ is the L1-norm, the sum of the elements of the vector (Figure 1F). The lasso model was implemented via a “glmnet” R package, and the best lambda was determined by grid search. The lasso model was implemented on each given drug, respectively. The Pearson Correlation Coefficient (PCC) was calculated between experimentally tested results and predicted values, and drugs with PCC greater than 0.7 were kept for further analysis. According to the regression coefficient given by the lasso regression model, we selected the top 100 CpG islands as methylation sites related to drug effectiveness. The ENCODE database, which provides a wealth of data and clarifies the role of functional elements in the human genome (Ecker et al., 2012), was applied to check whether the identified methylation sites were located in the promoter region, enhancer region or TF binding region. Furthermore, the TRRUST2 database, the most comprehensive public database for literature-curated TF-target interactions in humans (Han et al., 2018), was introduced to test whether the methylation sites share loci with downstream gene’s TF binding region.

## Results

### Evaluation of the Contribution of DNA Methylation in Prediction of Drug Responses

**FIGURE 2:**
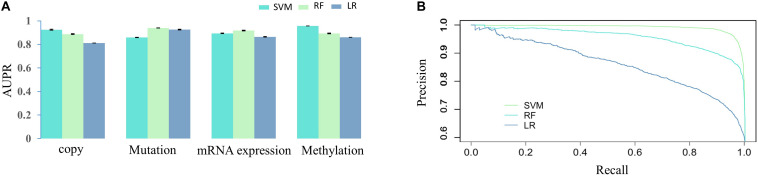
The AUPRs and PR curve on various data source. **(A)** AUPRs obtained by three classification algorithms (SVM, RF, and LR) based on different cancer omics data resources. **(B)** The PR curves obtained by three prediction models (SVM, RF, and LR) based on DNA methylation.

We firstly assessed the contribution of DNA methylation in prediction of drug responses and compared it with other cancer omics data resources. The AUPR was calculated through fivefold cross-validation based on each cancer omics data resource and is shown as the barplot in Figure 2. As we can see in Figure 2A, the SVM model using DNA methylation data performs best in SVM prediction model. As for RF and LR models, the best performance is achieved by using mutation data. Overall, no matter which classification model is used, we can see the predictive performance of DNA methylation is comparable to those of other cancer omics data. Figure 2B shows the PR curve of three prediction models based on methylation data, respectively. It can be concluded that the SVM outperforms RF and LR by achieving best AUPR. We also provided the AUC (Lobo, 2007) obtained on different data resources and different classification models in Supplementary Material. The methylation data achieved AUC of 0.94 ± 0.0017, 0.99 ± 0.0004, and 0.84 ± 0.0003 for SVM, RF, and LR, respectively, which were comparable with other data resources (Supplementary Table 1). These results together indicate the important role of DNA methylation in prediction of drug responses. In Supplementary Material, we provided *P* values obtained by different predictive models based on different data resources. Most of these *P-*values are less than 0.01, except for methylation versus copy number in the RF model and methylation versus RNA-seq in the LR model (Supplementary Table 2). Therefore, we conclude that DNA methylation data could be used as an effective data resource to predict the responses of cancer cell lines to anticancer drugs.

### Validation of the Effectiveness of DNA Methylation Through an Independent Test

To verify the contribution of DNA methylation in drug response prediction, we introduced DNA methylation data and experimental drug screening results from the CCLE database and applied them as the independent test data. Specifically, we trained the drug response model through the GDSC methylation data and experimental drug screening results and applied that model to predict the cell-drug relationships in CCLE based on methylation data. The active area value (the area over the dose-response curve) was introduced to quantify drug sensitivity (Supplementary Figure 1). We presented the RF prediction results in Supplementary Material (Supplementary Figure 2). The PCC between RF prediction scores and experimental results is 0.558, and the predictive score for the sensitive group and the resistance group are significantly different (*P* < 2.2e-16). These results suggest the great generalization ability of prediction model based on DNA methylation data.

### Discussion of the Mechanism of DNA Methylation in Regulation of Drug Effectiveness

**FIGURE 3:**
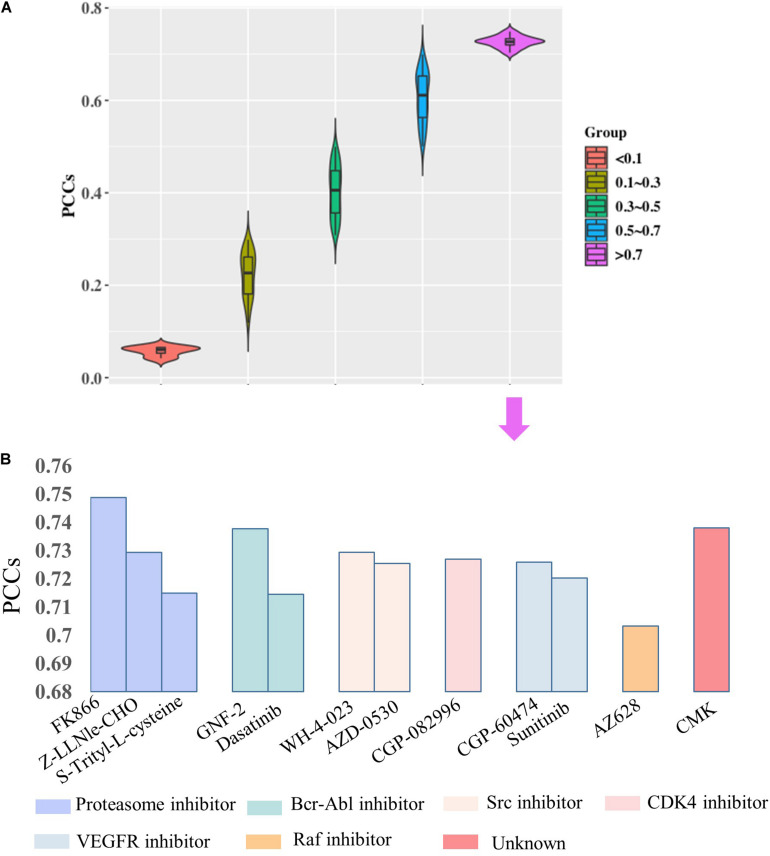
Correlation coefficients of experimentally tested results and predicted values for 209 drugs. **(A)** The violin diagram shows the PCCs between experimental results and the predictions. The PCCs in the five groups of “<0.1,” “0.1–0.3,” “0.3–0.5,” “0.5–0.7,” and “>0.7” are shown. **(B)** The barplot shows the PCCs for 12 drugs with PCCs greater than 0.7. The PCCs values for different types of inhibitors, including Proteasome inhibitor, Bcr-Abl inhibitor, Src inhibitor, CDK4 inhibitor, VEGFR inhibitor, and Raf inhibitor, are shown with different colors.

Then, we would like to discuss the mechanism of DNA methylation in regulation of drug effectiveness. To this end, we introduced a lasso regression model that performs both variable selection and regularization for improving the prediction accuracy and enhancing the interpretability of the statistical model (Fadil and William, 1986). By taking methylation level of CpG islands as the regulators, and drug effectiveness (AUCDR) as the responses, the methylation sites that were closely related to drug effectiveness in a given drug were detected by lasso. The PCCs between experimentally tested results and predictive results for 209 drugs were shown in Figure 3. Figure 3A shows the distribution of PCCs in 209 drugs, which is mainly concentrated in the range of 0.3–0.5 and 0.5–0.7. Figure 3B shows the PCCs of 12 drugs with PCCs greater than 0.7, and the exact PCCs for these 12 drugs can be seen in Table 1. Therefore, the methylation sites related tp responses from these 12 drugs were discussed in the following subsection.

**TABLE 1:** PCCs values of 12 drugs.

**Drug name**	**PCCs**
Sunitinib	0.720
FK866	0.749
Z-LLNle-CHO	0.729
S-Trityl-L-cysteine	0.715
GNF-2	0.738
CMK	0.738
AZD-0530	0.725
WH-4-023	0.729
CGP-082996	0.727
CGP-606474	0.726
AZ628	0.703
Dasatinib	0.714

According to the regression coefficient of lasso model, the methylation sites closely related to these 12 drugs were screened out, and the top 100 CpG islands remained for further analysis. To discuss the regulatory roles of these methylation sites in drug effectiveness, ENCODE database was introduced to query the location information of them. After checking the TF binding sites (TFBS) information from the ENCODE database, we find that most of the selected methylation sites share the loci with TF binding region (Table 2). For example, the 100 methylation sites associated with Sunitinib response are located in the binding regions of 100 TFs. We performed the TFBS enrichment analysis on these methylation sites. Specifically, we randomly selected 100 fragments with average length of CpG Islands (about 2,000 bp) from non-coding region of the whole human genome. Then, we checked how many of these 100 fragments were located in TF binding region. We compared the frequency of predicted CpG Islands located in TFBS with this distribution function simulated by 1,000 repeats and calculated the *P-*value. As a result, *P-*values are smaller than 1e-26 for all 12 drugs. All these results indicate that the methylation sites closely related to drug effectiveness are enriched in the TFBS.

**TABLE 2:** The percentage of methylation sites located in TFBS and have corresponding TF target gene available in TRRUST2 database.

**Drug name**	**No. of methylation sites (%)**	**No. of TF (%)**	**Supplementary table**
Sunitinib	100	92	3,4
FK866	100	96	5,6
Z-LLNle-CHO	99	91	7,8
S-Trityl-L-cysteine	99	91	9,10
GNF-2	99	92	11,12
CMK	99	92	13,14
AZD-0530	99	90	15,16
WH-4-023	99	93	17,18
CGP-082996	99	92	19,20
CGP-606474	99	94	21,22
AZ628	98	91	23,24
Dasatinib	98	87	25,26

**FIGURE 4:**
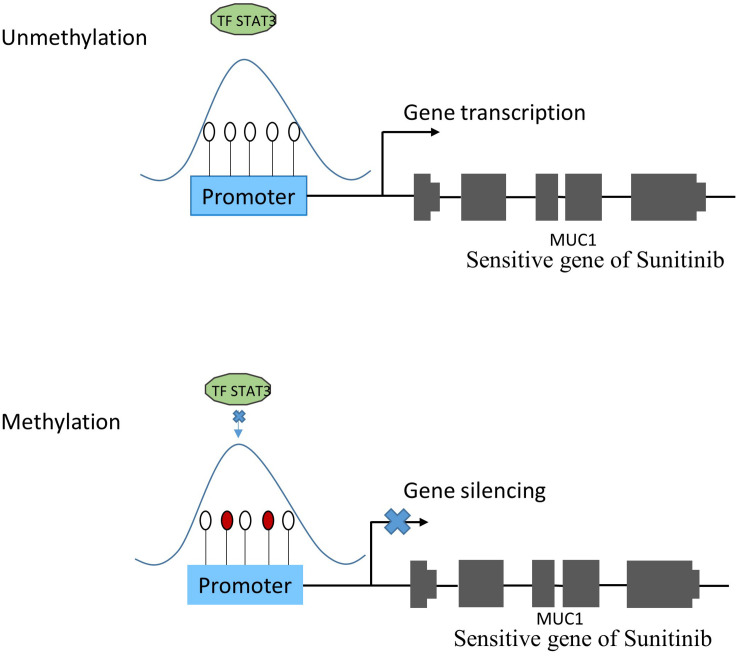
The relationship between methylation and gene expression. Under normal condition (subfigure A), TF *STAT3* binds the promoter region of *MUC1* gene that was proven to be sensitive to Sunitinib, and make it transcribed. However, when TF *STAT3* binding region was methylated (subfigure B), *STAT3* could not bind as usual, which might lead to *MUC1* gene silencing, thus affecting the sensitivity of Sunitinib.

The TRRUST2 database was further introduced to explore regulatory relationships between TFs and their target genes. As a result, among 100 methylation sites, 92 methylation sites share loci with TFBS, which have a regulatory target gene in TRRUST2. These results together indicate that methylation sites related to drug effectiveness share the loci with TFBS, and the variation in these methylation sites may interrupt the transcription regulated by corresponding TFs. That is, the variation in DNA methylation may block the normal binding of TFs, thus affect the normal transcription of their target genes that linked to drug effectiveness. Through a literature search, we found that the *MUC1* gene is related to the sensitivity of drug Sunitinib. It has been proved by experiments that the expression level of gene *MUC1* in renal cell carcinoma cell lines correlated to resistance to Sunitinib (Chen et al., 2018). Here, the lasso model for drug Sunitinib reveals the drug effectiveness related methylation site of chr1: 110880394-110880624. After database searching, it was found that this methylation site is located in the TF *STAT3* binding region, while the TF *STAT3* regulates the transcription of *MUC1* gene (Figure 4).

From the above analysis, we attempted to provide a possible mechanism of DNA methylation in regulation of drug effectiveness. Then, we checked the relationship between DNA methylation and downstream gene expression. Pearson correlation analysis was performed based on β values of methylation sites associated with drug response and expression values of downstream gene (Supplementary Figure 3). The good correlation relationship between DNA methylation and downstream gene expression is suggested. For instance, for drug “Dasatinib,” among 100 pairs of methylation sites and corresponding downstream gene, there are seven pairs of methylation sites and their downstream genes with PCCs larger than 0.5 and 42 pairs with PCCs larger than 0.3 and smaller than 0.5.

## Discussion

A systematic study of the relationship between cancer cells and anticancer therapies could inform early clinical trials of many new compounds. A series of efforts were adopted to improve the accuracy of prediction in our study. First, by assigning response value into three classes: sensitive, resistant, and unclear, and the “categorical” classifiers were introduced to detect whether a given cancer cell was sensitive or resistant to a specific anti-cancer therapy. Second, to overcome the heterogeneity of pharmacogenomic data, the similarity matrices were constructed to represent cancer cell lines and anti-cancer drugs. The purpose of replacing the original data with similarity matrix is to keep the samples number and feature number at the same scale, so as to attempt to avoid the overfitting. Third, three common classical classification models, namely SVM, RF, and LR, were introduced to assess the contribution of DNA methylation in prediction of drug responses. The results suggest that DNA methylation data performs best in the SVM model, and for RF and LR models, the prediction performance of DNA methylation is comparable to that of other data resources. In the previous work, a lot of research has been done on establishing drug response prediction using machine learning, mostly based on gene expression data. For example, Wang et al. (2016) used an SVM model to predict the drug response of mutation data, copy number and expression number from CCLE database, and found that the prediction value of mutation data was the best; Riddick et al. (2011) used an RF model to fit the drug IC50 with underlying gene expression and has been shown to successfully predict drug response, outperformed other methods based on differential gene expression. We are committed to optimize and strengthen the models in drug responses, mainly because the cost of drug design and time consuming clinical trials are the major costs of cancer treatment, while the application of machine learning can greatly reduce the cost.

In this paper, we also attempted to validate the role of DNA methylation in prediction of drug response by an independent data test. The RF model based on DNA methylation data indicates that the predicted results correlate well with the experimental ones (PCC = 0.556).

This suggests that DNA methylation can be used as an informative data resource to predict drug response.

Here, methylation sites associated with sensitivity or resistance to anticancer drugs are predicted based on the lasso regression model. A total of 12 drugs are found to have good correlations between predictions and experimental drug screening results. The database search indicates that almost all the methylation sites associated with the drug effectiveness are located in the TFBS (Table 2). The further enrichment analysis indicates that the methylation sites closely related to drug effectiveness are enriched in TFBS (*p*-value less than 1e-26). Therefore, we hypothesize that DNA methylation may affect the normal binding of TF, and then change the expression level of their target genes that are linked with drug responses. In the prediction of methylation sites related to Sunitinib, the binding region of TF *STAT3* is found to contain the methylation site (chr1: 110880394-110880624) that related to drug effectiveness, and TF *STAT3* target gene *MUC1* is related to drug Sunitinib response. These results suggest the possible regulation role of methylation site in drug effectiveness, that is, methylation may interrupt the normal binding of TF to its target gene that are related to drug response. Therefore, our future work will be designed to discover more genes that have been experimentally verified to be related to drug response and to further verify our hypothesis.

In addition, we performed correlation analysis between the selected methylation sites and their downstream genes, and the results show 42% pairs of methylation sites and their downstream genes have PCCs larger than 0.3. Furthermore, the previous studies suggested the correlation between CpG Islands shores and downstream genes (Irizarry et al., 2009). Thus, we also did a correlation analysis between methylation of the CpG Islands shores and the expression of downstream genes. Here, we find a total of 159 pairs of CpG Islands shores and downstream genes with both methylation β value and expression available, and the results show that 18 pairs of them has PCCs larger than 0.3.

In summary, this study indicates the important role of DNA methylation in prediction of drug response, and reveals methylation sites related to drug effectiveness. The database and literature searches on those methylation sites offers a possible mechanism of DNA methylation in regulation of drug effectiveness. This information is helpful for people to further understand the regulation mechanism of drug responses to cancers.

## Data Availability Statement

All datasets presented in this study are included in the article/Supplementary Material.

## Author Contributions

RY was involved in literature review, data processing, and manuscript writing. YW was involved in important tasks such as guidance and constructive amendments to the manuscript. SC helped in the editing of the manuscript. All authors contributed to the article and approved the submitted version.

## Conflict of Interest

The authors declare that the research was conducted in the absence of any commercial or financial relationships that could be construed as a potential conflict of interest.
