# Supplementary material for: Computational Prediction of Drug Responses in Cancer Cell Lines From Cancer Omics and Detection of Drug Effectiveness Related Methylation Sites
Source: Front Genet. 2020 Aug 7;11:917. doi: 10.3389/fgene.2020.00917 (PMC7426400; doi:10.3389/fgene.2020.00917)
Supplement: Supplementary file 1 [file Data_Sheet_1.docx]

Supplementary Material

# Supplementary Figure


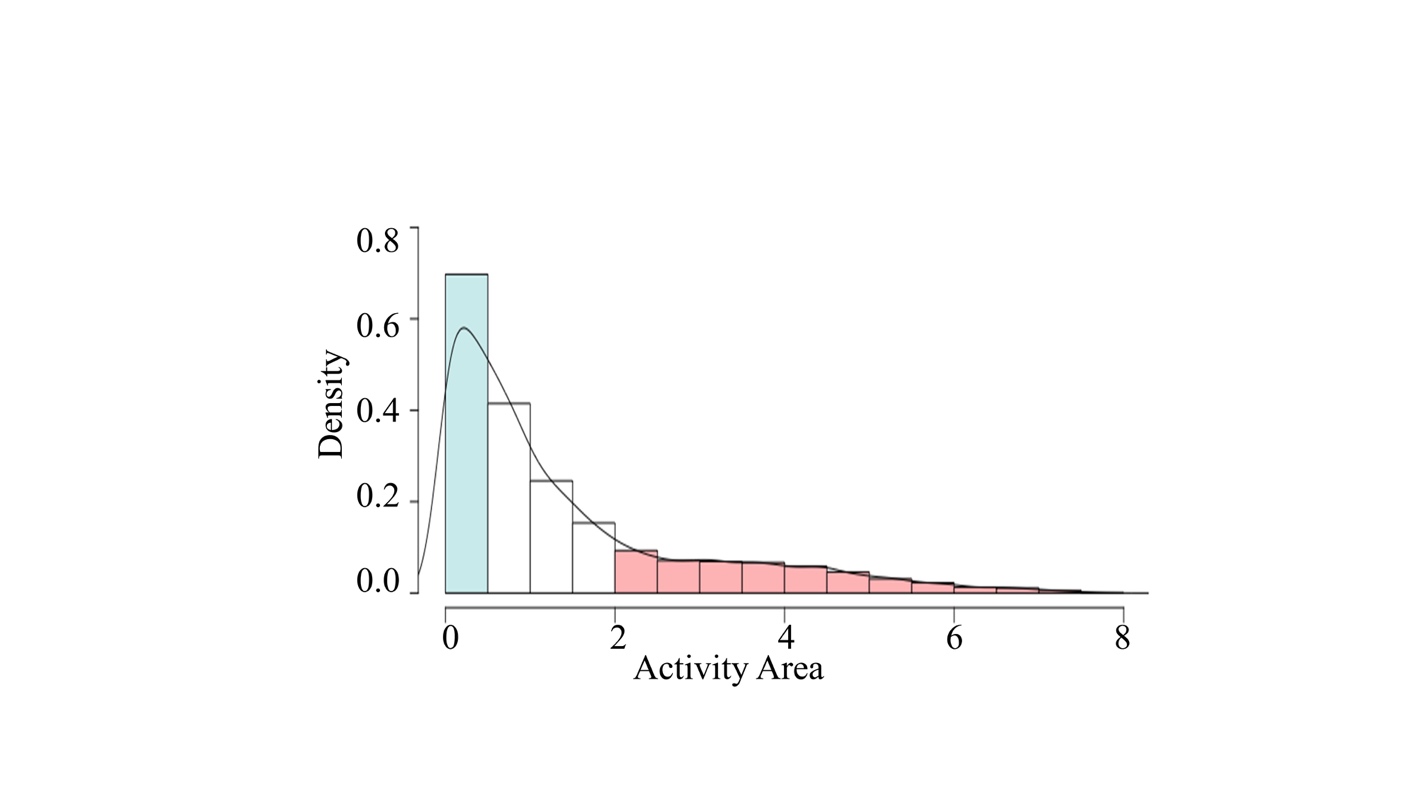


resistant

sensitive

**Supplementary Figure 1.** the distribution of experimentally tested Activity Area (area over the dose–response curves) indicate two phases of drug responses: resistant if experimentally tested Activity Area less than 0.5, and sensitive if experimentally tested Activity Area greater than 2. As a result, 6,894 resistant and 5,914 sensitive pairs of cancer cells and drugs were generated.


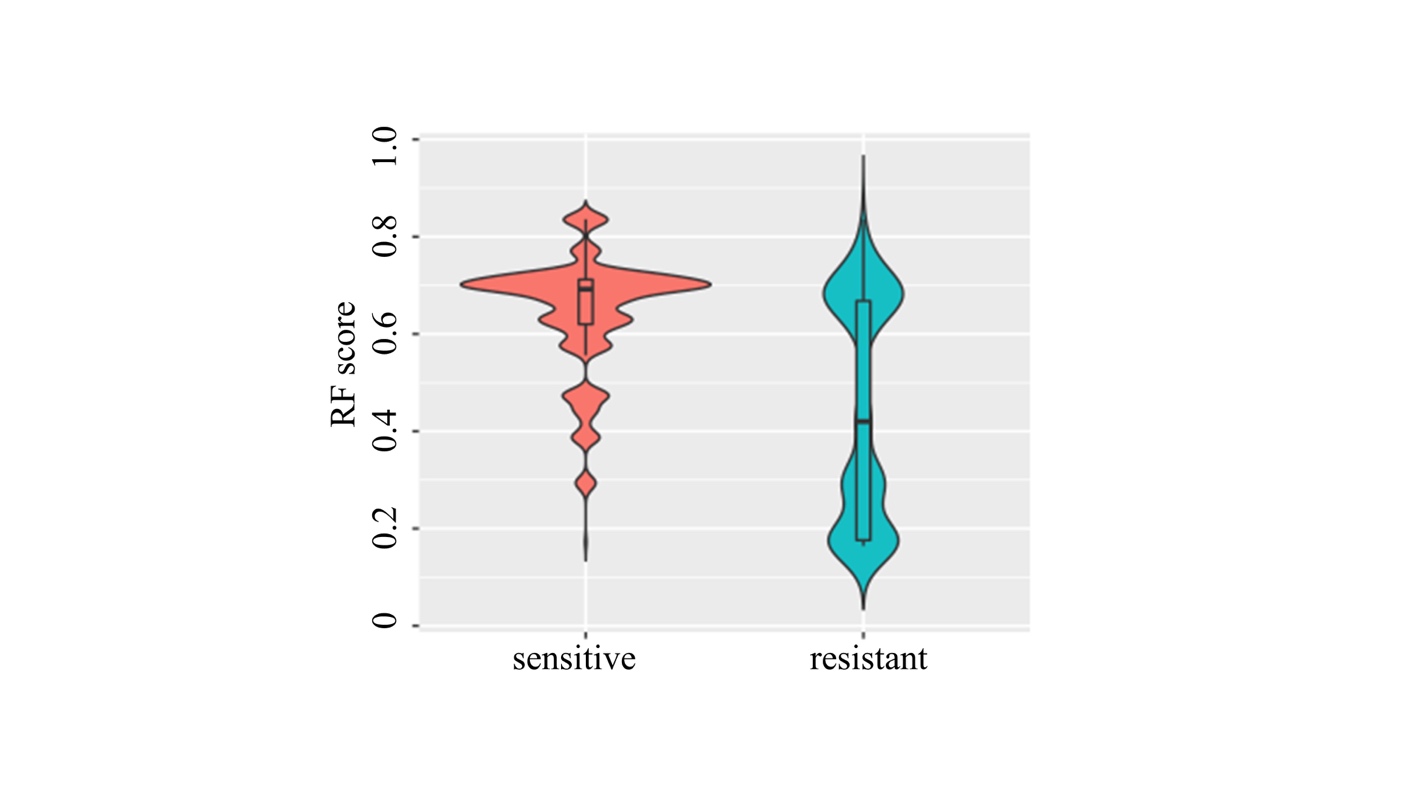


**Supplementary Figure 2.** An RF drug response model related to DNA methylation was constructed using an independent test set, and the predicted results were the score distribution of resistance and sensitivity.


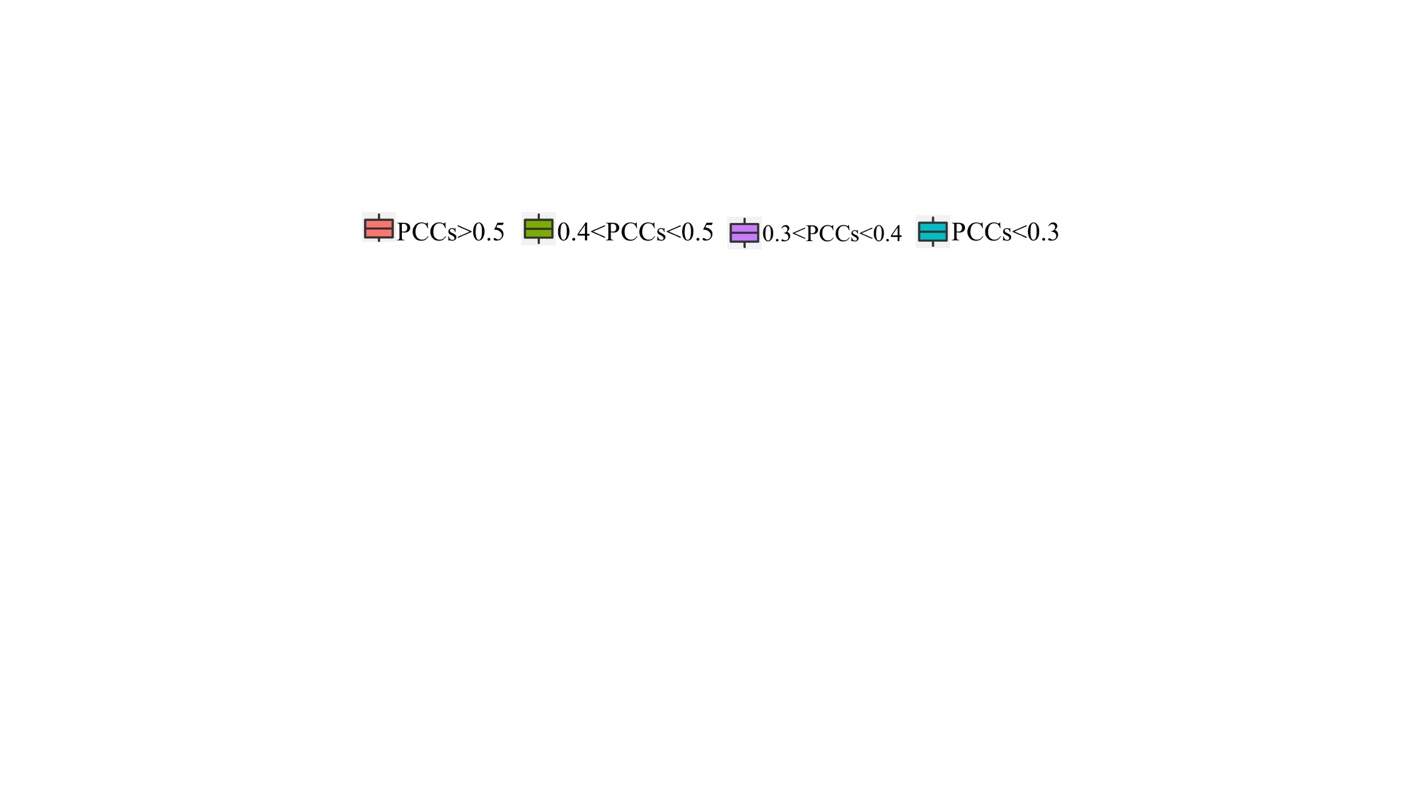

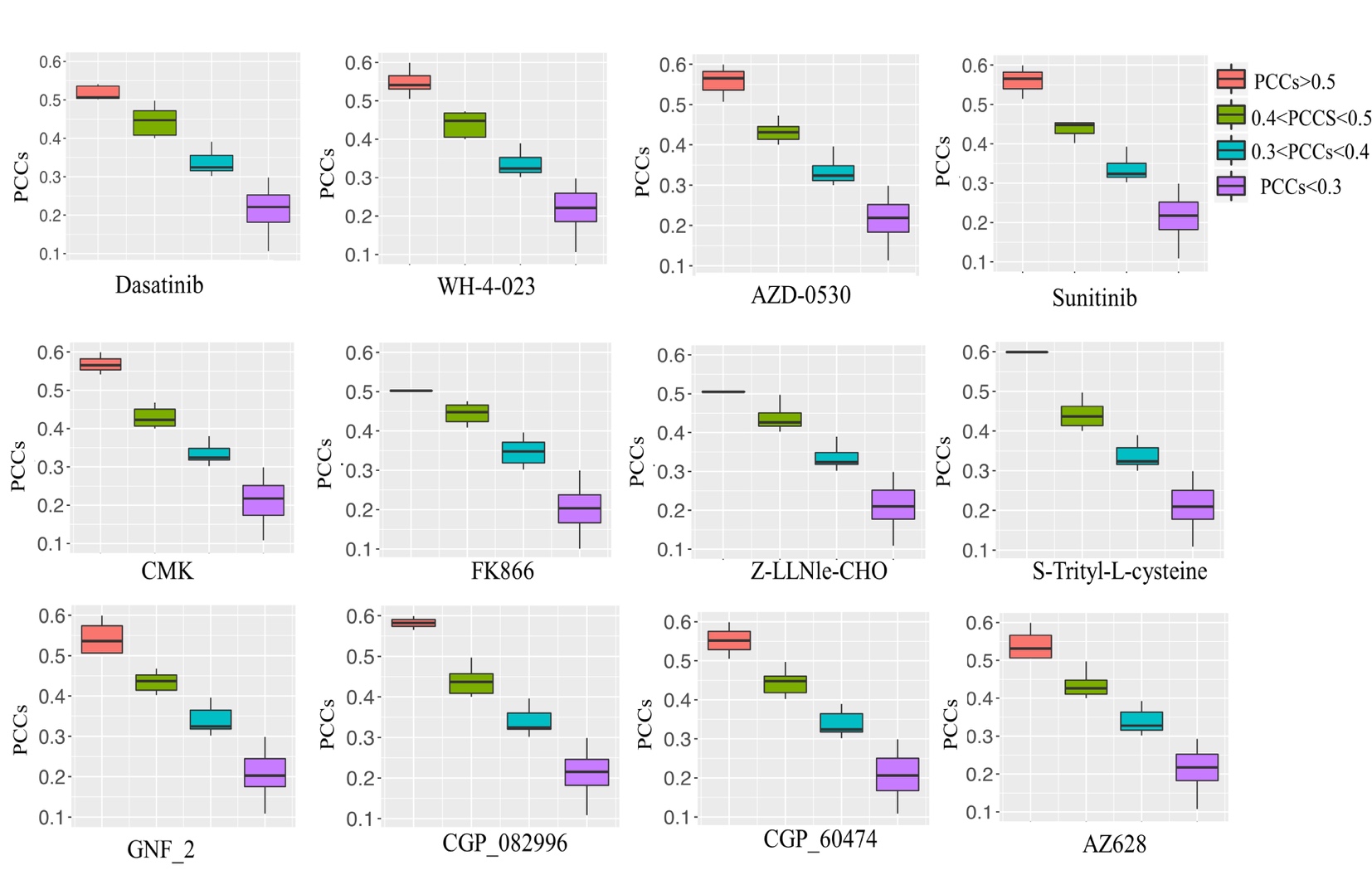


**Supplementary Figure 3**. Pearson correlation analysis was performed based on $\beta$ values of methylation sites associated with 12 anti-drug response and corresponding downstream gene expression values. It shows the distribution of PCCs values of each drug.

# Supplementary Tables

**Table 1.** AUC values of SVM、RF、LR models from different data sources.

| data source | SVM | RF | LR |
| --- | --- | --- | --- |
| copy | 0.93±0.003 | 0.98±0.0001 | 0.81±0.0004 |
| Mutation | 0.89±0.001 | 0.99±0.0002 | 0.94±0.001 |
| mRNA expression | 0.91±0.001 | 0.98±0.0002 | 0.85±0.002 |
| Methylation | 0.94±0.002 | 0.99±0.0004 | 0.84±0.0003 |

**Table 2.** P values obtained by comparing the drug response models based on different genomes.

|  | SVM | RF | LR |
| --- | --- | --- | --- |
| Met vs Mut | 1.75e-05 | 0.001149 | 0.0007626 |
| Met vs RNA | 0.0002378 | 0.001112 | 0.06091 |
| Met vs CN | 0.004963 | 0.1492 | 6.34e-06 |
| Mut vs RNA | 5.91e-05 | 0.005267 | 4.99e-05 |
| Mut vs CN | 0.0003806 | 0.001405 | 0.000173 |
| RNA vs CN | 0.001177 | 0.0008113 | 0.0001546 |

**Table 3.** Predicted methylation sites related to Sunitinib drug sensitivity, 100 of which were located in the transcription factor binding region.

| chr1:10092532-10093404 | chr1:110880394-110880624 | chr1:11714217-11715304 | chr1:145470241-145470673 |
| --- | --- | --- | --- |
| chr1:154973250-154974358 | chr1:1590073-1590942 | chr1:180991719-180992149 | chr1:19536151-19537046 |
| chr1:22778236-22778817 | chr1:24306363-24307017 | chr1:26185472-26186356 | chr1:87380162-87381214 |
| chr1:95699725-95700142 | chr2:175112926-175113743 | chr2:183730808-183731357 | chr2:190539026-190539533 |
| chr2:191878608-191879253 | chr2:20101004-20102067 | chr2:219263098-219265556 | chr2:230932468-230933550 |
| chr2:27440128-27440786 | chr3:184056419-184056671 | chr3:193310824-193311188 | chr3:196693765-196694200 |
| chr3:38206498-38207642 | chr3:9404683-9404938 | chr4:152020470-152021110 | chr4:25161781-25162290 |
| chr4:73934944-73935650 | chr4:76861089-76862426 | chr5:151150014-151152086 | chr5:71616007-71616345 |
| chr6:146284795-146285701 | chr6:151646668-151646958 | chr6:32811494-32811839 | chr6:36853590-36853927 |
| chr7:148395430-148397002 | chr7:7222020-7222974 | chr7:8473139-8475199 | chr7:99724482-99725808 |
| chr8:135724770-135725552 | chr8:144349048-144350070 | chr8:145742464-145743826 | chr8:22864324-22864588 |
| chr8:27471954-27472523 | chr8:28573597-28573840 | chr9:115983419-115984109 | chr9:37120050-37120985 |
| chr10:103603166-103603642 | chr10:104473399-104474711 | chr10:27444133-27444494 | chr10:99446696-99447400 |
| chr11:43333439-43333887 | chr11:45868742-45869413 | chr11:64001572-64002712 | chr11:65685100-65685364 |
| chr12:105724272-105725052 | chr12:109746826-109748378 | chr12:133337981-133338989 | chr12:5153012-5154346 |
| chr12:65563195-65564396 | chr13:111267780-111268778 | chr13:37574792-37575223 | chr13:39612417-39612930 |
| chr13:41345062-41345570 | chr13:77900504-77901140 | chr13:99228319-99229576 | chr14:73925150-73925435 |
| chr15:42066488-42067230 | chr15:52311220-52312180 | chr15:65321431-65321996 | chr16:2286600-2288073 |
| chr16:30006578-30007874 | chr16:56671937-56672530 | chr16:74734152-74734572 | chr17:1063942-1064304 |
| chr17:1394439-1394813 | chr17:34890466-34891480 | chr17:49337102-49338583 | chr17:55962573-55963229 |
| chr18:23713728-23713953 | chr18:59560494-59562016 | chr18:72999650-73000088 | chr19:1103722-1105758 |
| chr19:12807005-12807561 | chr19:17212479-17213332 | chr19:35454467-35455247 | chr19:42579935-42580588 |
| chr19:46366202-46366605 | chr19:51522004-51522803 | chr19:6425485-6425843 | chr20:1099078-1099561 |
| chr20:30311065-30311872 | chr20:36148603-36150136 | chr21:38362015-38362868 | chr21:46292709-46294744 |
| chr22:19841883-19843010 | chr22:24384134-24384405 | chr22:40766459-40767033 | chr22:43583502-43583830 |

**Table 4.** Methylation sites associated with Sunitinib drug sensitivity were located in 92 known transcription factor binding regions.

| ARID3A | ATF1 | ATF2 | ATF3 | BACH1 | BCL3 | BRCA1 | CBX8 |
| --- | --- | --- | --- | --- | --- | --- | --- |
| CEBPB | CEBPD | CHD4 | CREB1 | CREBBP | CTCF | CUX1 | E2F1 |
| E2F4 | E2F6 | EGR1 | ELF1 | ELK1 | ELK4 | EP300 | ESRRA |
| ETS1 | EZH2 | FOS | FOSL1 | FOSL2 | FOXA2 | FOXM1 | GABPA |
| GATA1 | GATA2 | GATA3 | HCFC1 | HDAC1 | HDAC2 | HNF4A | IRF1 |
| IRF3 | JUN | JUND | KAT2B | MAX | MAZ | MEF2A | MTA3 |
| MXI1 | MYBL2 | MYC | NCOR1 | NFATC1 | NFIC | NFYA | NFYB |
| NR2C2 | NR2F2 | NRF1 | PML | POU2F2 | RELA | REST | RFX5 |
| RUNX3 | RXRA | SIN3A | SMARCA4 | SMARCB1 | SP1 | SP4 | SPI1 |
| SREBF1 | SREBF2 | SRF | STAT1 | STAT2 | STAT3 | STAT5A | SUZ12 |
| TAF1 | TAL1 | TBP | TCF12 | TCF7L2 | TEAD4 | TRIM28 | USF1 |
| USF2 | YY1 | ZBTB7A | ZNF143 |  |  |  |  |

**Table 5.** Predicted methylation sites related to FK866 drug sensitivity, 100 of which were located in the transcription factor binding region.

| chr1:156022962-156025174 | chr1:167189130-167191361 | chr1:207925171-207925964 | chr1:225997646-225998233 |
| --- | --- | --- | --- |
| chr1:227751409-227751635 | chr1:7843915-7845104 | chr2:11295559-11296006 | chr2:187350497-187351609 |
| chr2:227700313-227701115 | chr2:241508424-241508627 | chr2:42396301-42396933 | chr2:54086776-54087266 |
| chr2:85197445-85199321 | chr2:96971125-96971556 | chr3:10290888-10291111 | chr3:156877175-156878364 |
| chr3:32544018-32544325 | chr3:49141934-49142373 | chr4:37828144-37828945 | chr4:48781331-48782339 |
| chr5:133707020-133707892 | chr5:169659830-169660229 | chr5:180670373-180670996 | chr5:7868863-7869840 |
| chr6:10882926-10883149 | chr6:117803497-117804671 | chr6:123110288-123110878 | chr6:160511936-160512465 |
| chr6:42419510-42419743 | chr6:44191278-44191872 | chr6:7312811-7313656 | chr7:100209594-100210655 |
| chr7:102311947-102312332 | chr7:105924650-105925975 | chr7:148395430-148397002 | chr7:150676178-150676585 |
| chr7:98741205-98741990 | chr9:116037549-116038229 | chr9:127951391-127952263 | chr9:128023933-128024596 |
| chr9:134378193-134378792 | chr9:140093882-140095686 | chr9:33263781-33265118 | chr10:104153729-104155557 |
| chr10:120101564-120101937 | chr10:120966127-120967623 | chr10:75541557-75542206 | chr10:81966989-81967517 |
| chr11:6947520-6948212 | chr11:77899564-77899990 | chr12:104350503-104351069 | chr12:12419679-12420541 |
| chr12:133337981-133338989 | chr12:6729248-6730310 | chr12:97300802-97301287 | chr13:40229743-40230181 |
| chr13:50570375-50571643 | chr14:21737406-21738094 | chr14:50086955-50088261 | chr14:59654630-59656330 |
| chr15:30918312-30918565 | chr15:93447556-93447823 | chr16:24550618-24551723 | chr16:4852117-4853176 |
| chr16:619805-620195 | chr16:67752849-67753446 | chr17:13503829-13505559 | chr17:27717135-27718037 |
| chr17:27915718-27916957 | chr17:40306909-40307263 | chr17:41150292-41150649 | chr17:42200521-42201543 |
| chr17:76183077-76183449 | chr17:76210129-76210627 | chr17:8339002-8339960 | chr18:21033071-21033657 |
| chr18:43753303-43755104 | chr18:56530395-56531288 | chr18:657401-658745 | chr18:72923054-72924182 |
| chr19:10676267-10677064 | chr19:11200919-11201686 | chr19:14168142-14168512 | chr19:15235912-15236631 |
| chr19:30097065-30097279 | chr19:37018919-37019811 | chr19:42811013-42811219 | chr19:45594397-45595007 |
| chr19:47126782-47127478 | chr19:48894463-48894927 | chr19:531114-532896 | chr19:55791274-55791818 |
| chr19:55918699-55919816 | chr19:56111498-56112063 | chr19:9785480-9785806 | chr20:30135076-30135292 |
| chr21:33765013-33765300 | chr21:34143453-34144141 | chr21:45758975-45759525 | chr22:50246590-50248143 |

**Table 6.** Methylation sites associated with FK866 drug sensitivity were located in 96 known transcription factor binding regions.

| ARID3A | ATF1 | ATF2 | ATF3 | BACH1 | BCL3 | BRCA1 | CBX8 |
| --- | --- | --- | --- | --- | --- | --- | --- |
| CEBPB | CEBPD | CHD4 | CREB1 | CREBBP | CTCF | CUX1 | E2F1 |
| E2F4 | E2F6 | EGR1 | ELF1 | ELK1 | ELK4 | EP300 | ESRRA |
| ETS1 | EZH2 | FOS | FOSL2 | FOXA1 | FOXA2 | FOXM1 | FOXP2 |
| GABPA | GATA1 | GATA2 | GATA3 | HCFC1 | HDAC1 | HDAC2 | HNF4A |
| IKZF1 | IRF1 | IRF3 | JUN | JUND | KAT2B | MAX | MAZ |
| MTA3 | MXI1 | MYBL2 | MYC | NCOR1 | NFATC1 | NFIC | NFYA |
| NFYB | NR2C2 | NR2F2 | NR3C1 | NRF1 | PML | POU2F2 | PRDM1 |
| RELA | REST | RFX5 | RXRA | SIN3A | SMARCB1 | SP1 | SP2 |
| SP4 | SPI1 | SREBF1 | SREBF2 | SRF | STAT1 | STAT3 | STAT5A |
| SUZ12 | TAF1 | TAL1 | TBP | TCF12 | TCF3 | TCF7L2 | TEAD4 |
| TRIM28 | USF1 | USF2 | YY1 | ZBTB7A | ZEB1 | ZNF143 | ZNF217 |

**Table 7.** Predicted methylation sites related to Z-LLNle-CHO drug sensitivity, 99 of which were located in the transcription factor binding region.

| chr1:10092532-10093404 | chr1:110880394-110880624 | chr1:11714217-11715304 | chr1:145470241-145470673 |
| --- | --- | --- | --- |
| chr1:154531054-154531565 | chr1:154973250-154974358 | chr1:180991719-180992149 | chr1:19536151-19537046 |
| chr1:21978101-21978764 | chr1:247241579-247242201 | chr1:26185472-26186356 | chr1:51433762-51435505 |
| chr1:87380162-87381214 | chr1:95699725-95700142 | chr2:190539026-190539533 | chr2:230932468-230933550 |
| chr2:27440128-27440786 | chr3:101280545-101280952 | chr3:172468372-172468845 | chr3:184056419-184056671 |
| chr3:196693765-196694200 | chr3:38206498-38207642 | chr3:49377340-49377854 | chr3:71630573-71633217 |
| chr3:9404683-9404938 | chr4:73934944-73935650 | chr4:78978365-78979256 | chr5:151150014-151152086 |
| chr5:71616007-71616345 | chr6:110501609-110501821 | chr6:146284795-146285701 | chr6:149887317-149888252 |
| chr6:151646668-151646958 | chr6:32811494-32811839 | chr6:36853590-36853927 | chr7:148395430-148397002 |
| chr7:2353629-2354075 | chr7:43965806-43966400 | chr7:5085411-5086342 | chr7:7222020-7222974 |
| chr7:8473139-8475199 | chr8:144349048-144350070 | chr8:145742464-145743826 | chr8:22864324-22864588 |
| chr8:27471954-27472523 | chr9:115983419-115984109 | chr9:2717903-2718953 | chr9:37120050-37120985 |
| chr10:103603166-103603642 | chr10:27444133-27444494 | chr10:99446696-99447400 | chr11:45868742-45869413 |
| chr11:59383165-59383410 | chr11:64001572-64002712 | chr12:105724272-105725052 | chr12:109746826-109748378 |
| chr12:11802752-11802983 | chr12:133337981-133338989 | chr12:5153012-5154346 | chr12:53441384-53441706 |
| chr12:65563195-65564396 | chr13:25861234-25861794 | chr13:37574792-37575223 | chr13:41345062-41345570 |
| chr13:99228319-99229576 | chr14:73925150-73925435 | chr15:42066488-42067230 | chr15:49447339-49447576 |
| chr16:2286600-2288073 | chr16:30006578-30007874 | chr16:3332472-3333847 | chr16:431189-432810 |
| chr16:56671937-56672530 | chr16:66835271-66835790 | chr16:74734152-74734572 | chr16:89487760-89488158 |
| chr17:1063942-1064304 | chr17:1394439-1394813 | chr17:17715696-17716219 | chr17:30770960-30772137 |
| chr17:34890466-34891480 | chr17:42092143-42092432 | chr17:49337102-49338583 | chr18:59560494-59562016 |
| chr18:72999650-73000088 | chr19:1103722-1105758 | chr19:12807005-12807561 | chr19:35454467-35455247 |
| chr19:42579935-42580588 | chr19:46366202-46366605 | chr19:51522004-51522803 | chr19:6425485-6425843 |
| chr20:30311065-30311872 | chr20:33680055-33680334 | chr20:36148603-36150136 | chr21:38362015-38362868 |
| chr22:19841883-19843010 | chr22:24384134-24384405 | chr22:43583502-43583830 |  |

**Table 8.** Methylation sites associated with Z-LLNle-CHO drug sensitivity were located in 91 known transcription factor binding regions.

| ARID3A | ATF1 | ATF2 | BACH1 | BCL3 | BRCA1 | CBX8 | CEBPB |
| --- | --- | --- | --- | --- | --- | --- | --- |
| CEBPD | CHD4 | CREB1 | CREBBP | CTCF | CUX1 | E2F1 | E2F4 |
| E2F6 | EGR1 | ELF1 | ELK1 | ELK4 | EP300 | ESRRA | ETS1 |
| EZH2 | FOS | FOSL1 | FOSL2 | FOXA2 | FOXM1 | GABPA | GATA1 |
| GATA2 | GATA3 | HCFC1 | HDAC1 | HDAC2 | HNF4A | IKZF1 | IRF1 |
| IRF3 | JUN | JUND | KAT2B | MAX | MAZ | MTA3 | MXI1 |
| MYBL2 | MYC | NCOR1 | NFATC1 | NFIC | NFYA | NFYB | NR2C2 |
| NR2F2 | NRF1 | PAX5 | PML | RELA | REST | RFX5 | RUNX3 |
| RXRA | SIN3A | SMARCA4 | SMARCB1 | SP1 | SP2 | SP4 | SPI1 |
| SREBF1 | SREBF2 | SRF | STAT1 | STAT3 | STAT5A | SUZ12 | TAF1 |
| TAL1 | TBP | TCF12 | TCF7L2 | TEAD4 | TRIM28 | USF1 | USF2 |
| YY1 | ZBTB7A | ZNF143 |  |  |  |  |  |

**Table 9.** Predicted methylation sites related to S-Trityl-L-cysteine drug sensitivity, 99 of which were located in the transcription factor binding region.

| chr1:10092532-10093404 | chr1:110880394-110880624 | chr1:11714217-11715304 | chr1:145470241-145470673 |
| --- | --- | --- | --- |
| chr1:154531054-154531565 | chr1:154973250-154974358 | chr1:180991719-180992149 | chr1:19536151-19537046 |
| chr1:219347109-219347572 | chr1:247241579-247242201 | chr1:26185472-26186356 | chr1:28052145-28053002 |
| chr1:38061428-38061740 | chr1:45139770-45140584 | chr1:87380162-87381214 | chr1:95699725-95700142 |
| chr2:183730808-183731357 | chr2:190539026-190539533 | chr2:191878608-191879253 | chr3:184056419-184056671 |
| chr3:38206498-38207642 | chr3:49377340-49377854 | chr3:9404683-9404938 | chr4:73934944-73935650 |
| chr4:76861089-76862426 | chr4:78978365-78979256 | chr5:151150014-151152086 | chr6:110501609-110501821 |
| chr6:149887317-149888252 | chr6:151646668-151646958 | chr6:32811494-32811839 | chr7:148395430-148397002 |
| chr7:2353629-2354075 | chr7:43965806-43966400 | chr7:5085411-5086342 | chr7:7222020-7222974 |
| chr7:8473139-8475199 | chr8:145742464-145743826 | chr8:22864324-22864588 | chr8:27471954-27472523 |
| chr9:115983419-115984109 | chr9:130742515-130743486 | chr9:2717903-2718953 | chr10:103603166-103603642 |
| chr10:27444133-27444494 | chr10:75531970-75532817 | chr10:99446696-99447400 | chr11:45868742-45869413 |
| chr11:59383165-59383410 | chr11:64001572-64002712 | chr11:65685100-65685364 | chr12:105724272-105725052 |
| chr12:109746826-109748378 | chr12:11802752-11802983 | chr12:12509911-12510428 | chr12:133337981-133338989 |
| chr12:14927291-14928023 | chr12:5153012-5154346 | chr12:53441384-53441706 | chr12:65563195-65564396 |
| chr13:25861234-25861794 | chr13:37574792-37575223 | chr13:41345062-41345570 | chr13:91999541-92001441 |
| chr13:99228319-99229576 | chr14:73925150-73925435 | chr15:65321431-65321996 | chr16:2286600-2288073 |
| chr16:30006578-30007874 | chr16:3332472-3333847 | chr16:56671937-56672530 | chr16:66835271-66835790 |
| chr16:74734152-74734572 | chr17:1063942-1064304 | chr17:1394439-1394813 | chr17:30770960-30772137 |
| chr17:34890466-34891480 | chr17:4634567-4635230 | chr17:49337102-49338583 | chr17:8151126-8151520 |
| chr18:46474931-46479364 | chr18:59560494-59562016 | chr19:1103722-1105758 | chr19:12807005-12807561 |
| chr19:42579935-42580588 | chr19:46236428-46236957 | chr19:46366202-46366605 | chr19:51522004-51522803 |
| chr19:6425485-6425843 | chr20:30311065-30311872 | chr20:32254811-32255989 | chr20:36148603-36150136 |
| chr20:44539729-44540099 | chr20:47804353-47805160 | chr21:38362015-38362868 | chr22:19841883-19843010 |
| chr22:24384134-24384405 | chr22:40766459-40767033 | chr22:43583502-43583830 |  |

**Table 10.** Methylation sites associated with S-Trityl-L-cysteine drug sensitivity were located in 91 known transcription factor binding regions.

| ARID3A | ATF1 | ATF2 | BACH1 | BCL3 | BRCA1 | CBX8 | CEBPB |
| --- | --- | --- | --- | --- | --- | --- | --- |
| CEBPD | CHD4 | CREB1 | CREBBP | CTCF | CUX1 | E2F1 | E2F4 |
| E2F6 | EGR1 | ELF1 | ELK1 | ELK4 | EP300 | ESRRA | ETS1 |
| EZH2 | FOS | FOSL1 | FOSL2 | FOXA2 | FOXM1 | GABPA | GATA1 |
| GATA2 | GATA3 | HCFC1 | HDAC1 | HDAC2 | HNF4A | IRF1 | IRF3 |
| JUN | JUND | KAT2B | MAX | MAZ | MTA3 | MXI1 | MYBL2 |
| MYC | NCOR1 | NFATC1 | NFIC | NFYA | NFYB | NR2C2 | NR2F2 |
| NRF1 | PML | RELA | REST | RFX5 | RUNX3 | RXRA | SIN3A |
| SMARCA4 | SMARCB1 | SP1 | SP2 | SP4 | SPI1 | SREBF1 | SREBF2 |
| SRF | STAT1 | STAT2 | STAT3 | STAT5A | SUZ12 | TAF1 | TAL1 |
| TBP | TCF12 | TCF7L2 | TEAD4 | TRIM28 | USF1 | USF2 | YY1 |
| ZBTB7A | ZEB1 | ZNF143 |  |  |  |  |  |

**Table 11.** Predicted methylation sites related to GNF-2 drug sensitivity, 99 of which were located in the transcription factor binding region.

| chr1:10092532-10093404 | chr1:11714217-11715304 | chr1:154531054-154531565 | chr1:154973250-154974358 |
| --- | --- | --- | --- |
| chr1:180991719-180992149 | chr1:19536151-19537046 | chr1:214161197-214161415 | chr1:21978101-21978764 |
| chr1:247241579-247242201 | chr1:26185472-26186356 | chr1:87380162-87381214 | chr1:95699725-95700142 |
| chr2:190539026-190539533 | chr2:230932468-230933550 | chr3:101280545-101280952 | chr3:172468372-172468845 |
| chr3:184056419-184056671 | chr3:38206498-38207642 | chr3:49377340-49377854 | chr4:151500763-151501299 |
| chr4:57521621-57522703 | chr4:73934944-73935650 | chr4:78978365-78979256 | chr5:151150014-151152086 |
| chr5:71616007-71616345 | chr6:146284795-146285701 | chr6:151646668-151646958 | chr6:32811494-32811839 |
| chr6:36853590-36853927 | chr7:148395430-148397002 | chr7:2353629-2354075 | chr7:43965806-43966400 |
| chr7:5085411-5086342 | chr7:7222020-7222974 | chr7:8473139-8475199 | chr8:144349048-144350070 |
| chr8:145742464-145743826 | chr8:22864324-22864588 | chr8:27471954-27472523 | chr9:115983419-115984109 |
| chr9:130742515-130743486 | chr9:134378193-134378792 | chr9:26956301-26956770 | chr9:37120050-37120985 |
| chr10:103603166-103603642 | chr10:27444133-27444494 | chr11:45868742-45869413 | chr11:59383165-59383410 |
| chr11:64001572-64002712 | chr11:65685100-65685364 | chr11:842293-843396 | chr12:105724272-105725052 |
| chr12:109746826-109748378 | chr12:11802752-11802983 | chr12:118499103-118499473 | chr12:133337981-133338989 |
| chr12:47629034-47629680 | chr12:50297580-50297988 | chr12:5153012-5154346 | chr12:53441384-53441706 |
| chr12:65563195-65564396 | chr13:25861234-25861794 | chr13:37574792-37575223 | chr13:41345062-41345570 |
| chr13:77900504-77901140 | chr13:99228319-99229576 | chr14:73925150-73925435 | chr15:42066488-42067230 |
| chr16:2286600-2288073 | chr16:30006578-30007874 | chr16:3332472-3333847 | chr16:431189-432810 |
| chr16:56671937-56672530 | chr16:66835271-66835790 | chr16:74734152-74734572 | chr16:89487760-89488158 |
| chr17:1063942-1064304 | chr17:1394439-1394813 | chr17:17715696-17716219 | chr17:30770960-30772137 |
| chr17:34890466-34891480 | chr17:42092143-42092432 | chr17:46125046-46126233 | chr17:7382266-7382710 |
| chr18:59560494-59562016 | chr18:72999650-73000088 | chr19:1103722-1105758 | chr19:12807005-12807561 |
| chr19:42579935-42580588 | chr19:46366202-46366605 | chr19:51522004-51522803 | chr19:6425485-6425843 |
| chr20:30311065-30311872 | chr20:36148603-36150136 | chr21:38362015-38362868 | chr22:19841883-19843010 |
| chr22:24384134-24384405 | chr22:40766459-40767033 | chr22:43583502-43583830 |  |

**Table 12.** Methylation sites associated with GNF-2 drug sensitivity were located in 92 known transcription factor binding regions.

| ARID3A | ATF1 | ATF2 | BACH1 | BCL3 | BRCA1 | CBX8 | CEBPB |
| --- | --- | --- | --- | --- | --- | --- | --- |
| CEBPD | CHD4 | CREB1 | CREBBP | CTCF | CUX1 | E2F1 | E2F4 |
| E2F6 | EGR1 | ELF1 | ELK1 | ELK4 | EP300 | ESRRA | ETS1 |
| EZH2 | FOS | FOSL1 | FOSL2 | FOXA1 | FOXA2 | FOXM1 | FOXP2 |
| GABPA | GATA1 | GATA2 | GATA3 | HCFC1 | HDAC1 | HDAC2 | HNF4A |
| IKZF1 | IRF1 | IRF3 | JUN | JUND | KAT2B | MAX | MAZ |
| MTA3 | MXI1 | MYBL2 | MYC | NCOR1 | NFATC1 | NFIC | NFYA |
| NFYB | NR2C2 | NR2F2 | NRF1 | PAX5 | PML | RELA | REST |
| RFX5 | RXRA | SIN3A | SMARCA4 | SMARCB1 | SP1 | SP2 | SP4 |
| SPI1 | SREBF1 | SREBF2 | SRF | STAT1 | STAT3 | STAT5A | SUZ12 |
| TAF1 | TAL1 | TBP | TCF12 | TCF7L2 | TEAD4 | TRIM28 | USF1 |
| USF2 | YY1 | ZBTB7A | ZNF143 |  |  |  |  |

**Table 13.** Predicted methylation sites related to CMK drug sensitivity, 99 of which were located in the transcription factor binding region.

| chr1:10092532-10093404 | chr1:110880394-110880624 | chr1:11714217-11715304 | chr1:154531054-154531565 |
| --- | --- | --- | --- |
| chr1:154973250-154974358 | chr1:180991719-180992149 | chr1:19536151-19537046 | chr1:21978101-21978764 |
| chr1:247241579-247242201 | chr1:26185472-26186356 | chr1:87380162-87381214 | chr1:94146321-94147504 |
| chr1:95699725-95700142 | chr2:190539026-190539533 | chr2:191878608-191879253 | chr2:27440128-27440786 |
| chr3:38206498-38207642 | chr3:49377340-49377854 | chr3:9404683-9404938 | chr4:73934944-73935650 |
| chr4:78978365-78979256 | chr5:151150014-151152086 | chr6:110501609-110501821 | chr6:146284795-146285701 |
| chr6:149887317-149888252 | chr6:151646668-151646958 | chr6:32811494-32811839 | chr6:36853590-36853927 |
| chr7:148395430-148397002 | chr7:43965806-43966400 | chr7:5085411-5086342 | chr7:7222020-7222974 |
| chr7:8473139-8475199 | chr8:144349048-144350070 | chr8:145742464-145743826 | chr8:22864324-22864588 |
| chr8:27471954-27472523 | chr9:108006467-108007921 | chr9:115983419-115984109 | chr9:130742515-130743486 |
| chr9:134378193-134378792 | chr9:2717903-2718953 | chr10:103603166-103603642 | chr10:27444133-27444494 |
| chr11:14665053-14666674 | chr11:45868742-45869413 | chr11:59383165-59383410 | chr11:64001572-64002712 |
| chr11:65685100-65685364 | chr12:105724272-105725052 | chr12:109746826-109748378 | chr12:11802752-11802983 |
| chr12:133337981-133338989 | chr12:47629034-47629680 | chr12:5153012-5154346 | chr12:65563195-65564396 |
| chr13:25861234-25861794 | chr13:37574792-37575223 | chr13:41345062-41345570 | chr13:77900504-77901140 |
| chr13:91999541-92001441 | chr13:99228319-99229576 | chr14:73925150-73925435 | chr14:74004122-74004683 |
| chr15:42066488-42067230 | chr16:2286600-2288073 | chr16:30006578-30007874 | chr16:3332472-3333847 |
| chr16:431189-432810 | chr16:56671937-56672530 | chr16:66835271-66835790 | chr16:74734152-74734572 |
| chr16:89487760-89488158 | chr17:1063942-1064304 | chr17:1394439-1394813 | chr17:17715696-17716219 |
| chr17:30770960-30772137 | chr17:34890466-34891480 | chr17:42092143-42092432 | chr17:49337102-49338583 |
| chr18:46474931-46479364 | chr18:59560494-59562016 | chr18:72999650-73000088 | chr19:1103722-1105758 |
| chr19:35454467-35455247 | chr19:42579935-42580588 | chr19:46236428-46236957 | chr19:46366202-46366605 |
| chr19:51522004-51522803 | chr19:54974175-54976819 | chr19:6425485-6425843 | chr20:30311065-30311872 |
| chr20:33680055-33680334 | chr20:36148603-36150136 | chr21:38362015-38362868 | chr22:19841883-19843010 |
| chr22:24384134-24384405 | chr22:40766459-40767033 | chr22:43583502-43583830 |  |

**Table 14.** Methylation sites associated with CMK drug sensitivity were located in 92 known transcription factor binding regions.

| ARID3A | ATF1 | ATF2 | BACH1 | BCL3 | BRCA1 | CBX8 | CEBPB |
| --- | --- | --- | --- | --- | --- | --- | --- |
| CEBPD | CHD4 | CREB1 | CREBBP | CTCF | CUX1 | E2F1 | E2F4 |
| E2F6 | EGR1 | ELF1 | ELK1 | ELK4 | EP300 | ESRRA | ETS1 |
| EZH2 | FOS | FOSL1 | FOSL2 | FOXA2 | FOXM1 | GABPA | GATA1 |
| GATA2 | GATA3 | HCFC1 | HDAC1 | HDAC2 | HNF4A | IRF1 | IRF3 |
| JUN | JUND | KAT2B | MAX | MAZ | MTA3 | MXI1 | MYBL2 |
| MYC | NCOR1 | NFATC1 | NFIC | NFYA | NFYB | NR2C2 | NR2F2 |
| NRF1 | PAX5 | PML | RELA | REST | RFX5 | RUNX3 | RXRA |
| SIN3A | SMARCA4 | SMARCB1 | SP1 | SP2 | SP4 | SPI1 | SREBF1 |
| SREBF2 | SRF | STAT1 | STAT2 | STAT3 | STAT5A | SUZ12 | TAF1 |
| TAL1 | TBP | TCF12 | TCF7L2 | TEAD4 | TRIM28 | USF1 | USF2 |
| YY1 | ZBTB7A | ZEB1 | ZNF143 |  |  |  |  |

**Table 15.** Predicted methylation sites related to AZD-0530 drug sensitivity, 99 of which were located in the transcription factor binding region.

| chr1:10092532-10093404 | chr1:10753773-10754176 | chr1:110880394-110880624 | chr1:11714217-11715304 |
| --- | --- | --- | --- |
| chr1:145470241-145470673 | chr1:154531054-154531565 | chr1:154973250-154974358 | chr1:180991719-180992149 |
| chr1:19536151-19537046 | chr1:214161197-214161415 | chr1:21978101-21978764 | chr1:22351917-22352194 |
| chr1:226249457-226251457 | chr1:24306363-24307017 | chr1:247241579-247242201 | chr1:26185472-26186356 |
| chr1:64058937-64059913 | chr2:190539026-190539533 | chr2:191878608-191879253 | chr2:230932468-230933550 |
| chr3:101280545-101280952 | chr3:11684192-11684930 | chr3:183959061-183959747 | chr3:38206498-38207642 |
| chr3:52279601-52280140 | chr4:57521621-57522703 | chr4:73934944-73935650 | chr4:78978365-78979256 |
| chr5:151150014-151152086 | chr5:179247784-179248711 | chr6:107349276-107349720 | chr6:110501609-110501821 |
| chr6:146284795-146285701 | chr6:151646668-151646958 | chr6:32811494-32811839 | chr6:36853590-36853927 |
| chr6:76311330-76312563 | chr7:108166211-108167077 | chr7:148395430-148397002 | chr7:5085411-5086342 |
| chr7:7222020-7222974 | chr7:74267121-74268083 | chr7:8473139-8475199 | chr8:22864324-22864588 |
| chr9:115983419-115984109 | chr9:134378193-134378792 | chr9:26956301-26956770 | chr10:103603166-103603642 |
| chr10:104473399-104474711 | chr10:120863620-120864216 | chr10:128973590-128974064 | chr10:75531970-75532817 |
| chr11:118977878-118978785 | chr11:406491-407871 | chr11:45868742-45869413 | chr11:64001572-64002712 |
| chr11:65685100-65685364 | chr11:842293-843396 | chr12:105724272-105725052 | chr12:109746826-109748378 |
| chr12:11802752-11802983 | chr12:133337981-133338989 | chr12:47629034-47629680 | chr12:50297580-50297988 |
| chr12:5153012-5154346 | chr12:53441384-53441706 | chr12:65563195-65564396 | chr13:25861234-25861794 |
| chr13:33161192-33161835 | chr13:37574792-37575223 | chr13:77900504-77901140 | chr13:99228319-99229576 |
| chr14:105218366-105218620 | chr14:21737406-21738094 | chr14:35098694-35099478 | chr14:73925150-73925435 |
| chr16:2286600-2288073 | chr16:30006578-30007874 | chr16:3332472-3333847 | chr16:74734152-74734572 |
| chr16:89487760-89488158 | chr17:1394439-1394813 | chr17:30770960-30772137 | chr17:34890466-34891480 |
| chr17:42092143-42092432 | chr17:49337102-49338583 | chr18:59560494-59562016 | chr19:1103722-1105758 |
| chr19:12807005-12807561 | chr19:46366202-46366605 | chr19:50059574-50060175 | chr19:50666147-50666514 |
| chr19:50713630-50714073 | chr19:54974175-54976819 | chr19:58038572-58039208 | chr19:6425485-6425843 |
| chr20:36148603-36150136 | chr21:38362015-38362868 | chr22:40766459-40767033 |  |

**Table 16.** Methylation sites associated with AZD-0530 drug sensitivity were located in 90 known transcription factor binding regions.

| ARID3A | ATF1 | ATF2 | BACH1 | BRCA1 | CBX8 | CEBPB | CEBPD |
| --- | --- | --- | --- | --- | --- | --- | --- |
| CHD4 | CREB1 | CREBBP | CTCF | CUX1 | E2F1 | E2F4 | E2F6 |
| EGR1 | ELF1 | ELK1 | ELK4 | EP300 | ESRRA | ETS1 | EZH2 |
| FOS | FOSL2 | FOXA1 | FOXA2 | FOXM1 | FOXP2 | GABPA | GATA1 |
| GATA2 | GATA3 | HCFC1 | HDAC1 | HDAC2 | HNF4A | IKZF1 | IRF1 |
| IRF3 | JUN | JUND | KAT2B | MAX | MAZ | MEF2A | MTA3 |
| MXI1 | MYBL2 | MYC | NCOR1 | NFATC1 | NFIC | NFYA | NFYB |
| NR2C2 | NR2F2 | NRF1 | PAX5 | PML | RELA | REST | RFX5 |
| RXRA | SIN3A | SMARCB1 | SP1 | SP4 | SPI1 | SREBF1 | SRF |
| STAT1 | STAT2 | STAT3 | STAT5A | SUZ12 | TAF1 | TAL1 | TBP |
| TCF12 | TCF7L2 | TEAD4 | TRIM28 | USF1 | USF2 | YY1 | ZBTB7A |
| ZEB1 | ZNF143 |  |  |  |  |  |  |

**Table 17.** Predicted methylation sites related to WH-4-023 drug sensitivity, 99 of which were located in the transcription factor binding region.

| chr1:11714217-11715304 | chr1:145713966-145714203 | chr1:154973250-154974358 | chr1:180991719-180992149 |
| --- | --- | --- | --- |
| chr1:185014369-185015220 | chr1:21978101-21978764 | chr1:24306363-24307017 | chr1:247241579-247242201 |
| chr1:28052145-28053002 | chr1:94146321-94147504 | chr1:948670-948894 | chr1:95699725-95700142 |
| chr2:175112926-175113743 | chr2:183730808-183731357 | chr2:190539026-190539533 | chr2:230932468-230933550 |
| chr3:172468372-172468845 | chr3:183959061-183959747 | chr3:38206498-38207642 | chr4:151500763-151501299 |
| chr4:73934944-73935650 | chr4:76861089-76862426 | chr5:132160904-132161809 | chr5:151150014-151152086 |
| chr5:71616007-71616345 | chr5:98263746-98265531 | chr6:151646668-151646958 | chr6:25726454-25726722 |
| chr6:32811494-32811839 | chr6:76311330-76312563 | chr7:148395430-148397002 | chr7:2353629-2354075 |
| chr7:43965806-43966400 | chr7:5085411-5086342 | chr7:7222020-7222974 | chr7:8473139-8475199 |
| chr8:144349048-144350070 | chr8:27471954-27472523 | chr9:130742515-130743486 | chr9:99145524-99145849 |
| chr10:103603166-103603642 | chr10:120863620-120864216 | chr10:123922850-123923542 | chr10:128973590-128974064 |
| chr11:118781060-118781732 | chr11:118977878-118978785 | chr11:45868742-45869413 | chr11:64001572-64002712 |
| chr11:65685100-65685364 | chr12:105724272-105725052 | chr12:109746826-109748378 | chr12:11802752-11802983 |
| chr12:131356094-131357228 | chr12:133337981-133338989 | chr12:47629034-47629680 | chr12:50297580-50297988 |
| chr12:53441384-53441706 | chr12:65563195-65564396 | chr13:37574792-37575223 | chr13:77900504-77901140 |
| chr13:99228319-99229576 | chr14:21737406-21738094 | chr14:37131181-37132785 | chr14:97263559-97264274 |
| chr15:42066488-42067230 | chr15:49447339-49447576 | chr15:93447556-93447823 | chr16:30006578-30007874 |
| chr16:3332472-3333847 | chr16:56671937-56672530 | chr16:89487760-89488158 | chr17:1063942-1064304 |
| chr17:1394439-1394813 | chr17:30770960-30772137 | chr17:34890466-34891480 | chr17:7342829-7344028 |
| chr18:23713728-23713953 | chr19:1103722-1105758 | chr19:12551471-12551877 | chr19:12807005-12807561 |
| chr19:18061195-18061473 | chr19:2474610-2476828 | chr19:42579935-42580588 | chr19:46236428-46236957 |
| chr19:46366202-46366605 | chr19:50059574-50060175 | chr19:50666147-50666514 | chr19:50713630-50714073 |
| chr19:632697-633678 | chr19:6425485-6425843 | chr19:8386082-8386972 | chr20:30311065-30311872 |
| chr20:32254811-32255989 | chr20:33680055-33680334 | chr20:36148603-36150136 | chr21:38362015-38362868 |
| chr22:19841883-19843010 | chr22:24384134-24384405 | chr22:40766459-40767033 |  |

**Table 18.** Methylation sites associated with WH-4-023 drug sensitivity were located in 93 known transcription factor binding regions.

| ARID3A | ATF1 | ATF2 | ATF3 | BACH1 | BCL3 | BRCA1 | CBX8 |
| --- | --- | --- | --- | --- | --- | --- | --- |
| CEBPB | CEBPD | CHD4 | CREB1 | CREBBP | CTCF | CUX1 | E2F1 |
| E2F4 | E2F6 | EGR1 | ELF1 | ELK1 | ELK4 | EP300 | ETS1 |
| EZH2 | FOS | FOSL1 | FOSL2 | FOXA2 | FOXM1 | GABPA | GATA1 |
| GATA2 | GATA3 | HCFC1 | HDAC1 | HDAC2 | HNF4A | IKZF1 | IRF1 |
| IRF3 | JUN | JUND | KAT2B | MAX | MAZ | MEF2A | MEF2C |
| MTA3 | MXI1 | MYBL2 | MYC | NCOR1 | NFATC1 | NFIC | NFYA |
| NFYB | NR2C2 | NR2F2 | NRF1 | PML | POU2F2 | PRDM1 | RELA |
| REST | RFX5 | RXRA | SIN3A | SMARCA4 | SMARCB1 | SP1 | SP4 |
| SPI1 | SREBF1 | SREBF2 | SRF | STAT1 | STAT2 | STAT3 | SUZ12 |
| TAF1 | TAL1 | TBP | TCF12 | TCF7L2 | TEAD4 | TRIM28 | USF1 |
| USF2 | YY1 | ZBTB7A | ZEB1 | ZNF143 |  |  |  |

**Table 19.** Predicted methylation sites related to CGP-082996 drug sensitivity, 99 of which were located in the transcription factor binding region.

| chr1:10092532-10093404 | chr1:110880394-110880624 | chr1:11714217-11715304 | chr1:145470241-145470673 |
| --- | --- | --- | --- |
| chr1:154531054-154531565 | chr1:154973250-154974358 | chr1:180991719-180992149 | chr1:19536151-19537046 |
| chr1:214161197-214161415 | chr1:21978101-21978764 | chr1:24306363-24307017 | chr1:247241579-247242201 |
| chr1:26185472-26186356 | chr1:87380162-87381214 | chr1:95699725-95700142 | chr2:190539026-190539533 |
| chr2:191878608-191879253 | chr2:230932468-230933550 | chr3:101280545-101280952 | chr3:184056419-184056671 |
| chr3:196693765-196694200 | chr3:38206498-38207642 | chr3:49377340-49377854 | chr3:9404683-9404938 |
| chr4:25161781-25162290 | chr4:57521621-57522703 | chr4:73934944-73935650 | chr4:78978365-78979256 |
| chr5:151150014-151152086 | chr6:146284795-146285701 | chr6:151646668-151646958 | chr6:32811494-32811839 |
| chr6:36853590-36853927 | chr7:148395430-148397002 | chr7:2353629-2354075 | chr7:5085411-5086342 |
| chr7:7222020-7222974 | chr7:8473139-8475199 | chr8:144349048-144350070 | chr8:145742464-145743826 |
| chr8:22864324-22864588 | chr8:27471954-27472523 | chr9:115983419-115984109 | chr9:130742515-130743486 |
| chr9:134378193-134378792 | chr9:26956301-26956770 | chr10:103603166-103603642 | chr10:27444133-27444494 |
| chr10:99446696-99447400 | chr11:45868742-45869413 | chr11:64001572-64002712 | chr11:65685100-65685364 |
| chr12:105724272-105725052 | chr12:109746826-109748378 | chr12:11802752-11802983 | chr12:133337981-133338989 |
| chr12:47629034-47629680 | chr12:5153012-5154346 | chr12:65563195-65564396 | chr13:25861234-25861794 |
| chr13:37574792-37575223 | chr13:41345062-41345570 | chr13:77900504-77901140 | chr13:99228319-99229576 |
| chr14:73925150-73925435 | chr14:74004122-74004683 | chr15:42066488-42067230 | chr16:2286600-2288073 |
| chr16:30006578-30007874 | chr16:3332472-3333847 | chr16:431189-432810 | chr16:56671937-56672530 |
| chr16:66835271-66835790 | chr16:74734152-74734572 | chr16:89487760-89488158 | chr17:1063942-1064304 |
| chr17:1394439-1394813 | chr17:17715696-17716219 | chr17:30770960-30772137 | chr17:34890466-34891480 |
| chr17:42092143-42092432 | chr17:49337102-49338583 | chr18:46474931-46479364 | chr18:59560494-59562016 |
| chr18:72999650-73000088 | chr19:1103722-1105758 | chr19:12807005-12807561 | chr19:42579935-42580588 |
| chr19:46366202-46366605 | chr19:51522004-51522803 | chr19:6425485-6425843 | chr20:30311065-30311872 |
| chr20:33680055-33680334 | chr20:36148603-36150136 | chr21:38362015-38362868 | chr22:19841883-19843010 |
| chr22:24384134-24384405 | chr22:40766459-40767033 | chr22:43583502-43583830 |  |

**Table 20.** Methylation sites associated with CGP-082996 drug sensitivity were located in 92 known transcription factor binding regions.

| ARID3A | ATF1 | ATF2 | BACH1 | BCL3 | BRCA1 | CBX8 | CEBPB |
| --- | --- | --- | --- | --- | --- | --- | --- |
| CEBPD | CHD4 | CREB1 | CREBBP | CTCF | CUX1 | E2F1 | E2F4 |
| E2F6 | EGR1 | ELF1 | ELK1 | ELK4 | EP300 | ESRRA | ETS1 |
| EZH2 | FOS | FOSL1 | FOSL2 | FOXA1 | FOXA2 | FOXM1 | FOXP2 |
| GABPA | GATA1 | GATA2 | GATA3 | HCFC1 | HDAC1 | HDAC2 | HNF4A |
| IRF1 | IRF3 | JUN | JUND | KAT2B | MAX | MAZ | MTA3 |
| MXI1 | MYBL2 | MYC | NCOR1 | NFATC1 | NFIC | NFYA | NFYB |
| NR2C2 | NR2F2 | NRF1 | PAX5 | PML | RELA | REST | RFX5 |
| RUNX3 | RXRA | SIN3A | SMARCA4 | SMARCB1 | SP1 | SP4 | SPI1 |
| SREBF1 | SREBF2 | SRF | STAT1 | STAT2 | STAT3 | STAT5A | SUZ12 |
| TAF1 | TAL1 | TBP | TCF12 | TCF7L2 | TEAD4 | TRIM28 | USF1 |
| USF2 | YY1 | ZBTB7A | ZNF143 |  |  |  |  |

**Table 21.** Predicted methylation sites related to CGP-60474 drug sensitivity, 99 of which were located in the transcription factor binding region.

| chr1:10092532-10093404 | chr1:110880394-110880624 | chr1:11714217-11715304 | chr1:145470241-145470673 |
| --- | --- | --- | --- |
| chr1:154531054-154531565 | chr1:154973250-154974358 | chr1:1590073-1590942 | chr1:165599563-165600574 |
| chr1:180991719-180992149 | chr1:19536151-19537046 | chr1:21978101-21978764 | chr1:24306363-24307017 |
| chr1:247241579-247242201 | chr1:26185472-26186356 | chr1:45139770-45140584 | chr1:87380162-87381214 |
| chr1:95699725-95700142 | chr2:190539026-190539533 | chr2:191878608-191879253 | chr2:27440128-27440786 |
| chr3:172468372-172468845 | chr3:184056419-184056671 | chr3:196693765-196694200 | chr3:38206498-38207642 |
| chr3:9404683-9404938 | chr4:73934944-73935650 | chr4:78978365-78979256 | chr5:151150014-151152086 |
| chr5:98263746-98265531 | chr6:146284795-146285701 | chr6:149887317-149888252 | chr6:151646668-151646958 |
| chr6:32811494-32811839 | chr6:36853590-36853927 | chr7:131012460-131013190 | chr7:148395430-148397002 |
| chr7:2353629-2354075 | chr7:5085411-5086342 | chr7:7222020-7222974 | chr7:8473139-8475199 |
| chr8:135724770-135725552 | chr8:144349048-144350070 | chr8:145742464-145743826 | chr8:22864324-22864588 |
| chr8:27471954-27472523 | chr9:115983419-115984109 | chr9:130742515-130743486 | chr9:2717903-2718953 |
| chr10:103603166-103603642 | chr10:27444133-27444494 | chr10:99446696-99447400 | chr11:14665053-14666674 |
| chr11:45868742-45869413 | chr11:59383165-59383410 | chr11:64001572-64002712 | chr11:65685100-65685364 |
| chr12:105724272-105725052 | chr12:109746826-109748378 | chr12:11802752-11802983 | chr12:133337981-133338989 |
| chr12:5153012-5154346 | chr12:53441384-53441706 | chr12:65563195-65564396 | chr13:25861234-25861794 |
| chr13:37574792-37575223 | chr13:41345062-41345570 | chr13:91999541-92001441 | chr13:99228319-99229576 |
| chr14:73925150-73925435 | chr15:49447339-49447576 | chr16:2286600-2288073 | chr16:30006578-30007874 |
| chr16:3332472-3333847 | chr16:431189-432810 | chr16:56671937-56672530 | chr16:66835271-66835790 |
| chr16:74734152-74734572 | chr17:1394439-1394813 | chr17:30770960-30772137 | chr17:34890466-34891480 |
| chr17:49337102-49338583 | chr18:23713728-23713953 | chr18:72923054-72924182 | chr18:72999650-73000088 |
| chr19:1103722-1105758 | chr19:35454467-35455247 | chr19:42579935-42580588 | chr19:46236428-46236957 |
| chr19:46366202-46366605 | chr20:1099078-1099561 | chr20:30311065-30311872 | chr20:36148603-36150136 |
| chr20:44539729-44540099 | chr20:47804353-47805160 | chr21:38362015-38362868 | chr22:19841883-19843010 |
| chr22:24384134-24384405 | chr22:40766459-40767033 | chr22:43583502-43583830 |  |

**Table 22.** Methylation sites associated with CGP-60474 drug sensitivity were located in 94 known transcription factor binding regions.

| ARID3A | ATF1 | ATF2 | BACH1 | BCL3 | BRCA1 | CBX8 | CEBPB |
| --- | --- | --- | --- | --- | --- | --- | --- |
| CEBPD | CHD4 | CREB1 | CREBBP | CTCF | CUX1 | E2F1 | E2F4 |
| E2F6 | EGR1 | ELF1 | ELK1 | ELK4 | EP300 | ESRRA | ETS1 |
| EZH2 | FOS | FOSL1 | FOSL2 | FOXA2 | FOXM1 | GABPA | GATA1 |
| GATA2 | GATA3 | HCFC1 | HDAC1 | HDAC2 | HNF4A | IKZF1 | IRF1 |
| IRF3 | JUN | JUND | KAT2B | MAX | MAZ | MEF2A | MTA3 |
| MXI1 | MYBL2 | MYC | NCOR1 | NFATC1 | NFIC | NFYA | NFYB |
| NR2C2 | NR2F2 | NRF1 | PML | POU2F2 | RELA | REST | RFX5 |
| RUNX3 | RXRA | SIN3A | SMARCA4 | SMARCB1 | SP1 | SP2 | SP4 |
| SPI1 | SREBF1 | SREBF2 | SRF | STAT1 | STAT2 | STAT3 | STAT5A |
| SUZ12 | TAF1 | TAL1 | TBP | TCF12 | TCF7L2 | TEAD4 | TRIM28 |
| USF1 | USF2 | YY1 | ZBTB7A | ZEB1 | ZNF143 | |  |

**Table 23.** Predicted methylation sites related to AZ628 drug sensitivity, 98 of which were located in the transcription factor binding region.

| chr1:10753773-10754176 | chr1:110880394-110880624 | chr1:117112960-117113883 | chr1:145713966-145714203 |
| --- | --- | --- | --- |
| chr1:154973250-154974358 | chr1:2005179-2005565 | chr1:214161197-214161415 | chr1:24306363-24307017 |
| chr1:247241579-247242201 | chr1:28052145-28053002 | chr2:131862756-131863444 | chr2:183730808-183731357 |
| chr2:190539026-190539533 | chr3:160472310-160474487 | chr3:172468372-172468845 | chr3:183959061-183959747 |
| chr3:184056419-184056671 | chr3:69134086-69134479 | chr4:154680774-154681377 | chr4:169239549-169239824 |
| chr4:73934944-73935650 | chr4:76861089-76862426 | chr4:79696707-79697958 | chr5:132361823-132362317 |
| chr5:140255158-140255450 | chr5:140531157-140532017 | chr5:145582725-145583511 | chr5:179247784-179248711 |
| chr6:149887317-149888252 | chr6:32811494-32811839 | chr6:84140412-84140998 | chr7:2353629-2354075 |
| chr7:5085411-5086342 | chr7:7222020-7222974 | chr7:8473139-8475199 | chr7:97910459-97911618 |
| chr7:99724482-99725808 | chr8:142237037-142237360 | chr8:144349048-144350070 | chr8:145017868-145018444 |
| chr8:22864324-22864588 | chr9:37120050-37120985 | chr9:37800565-37801563 | chr10:103603166-103603642 |
| chr10:104473399-104474711 | chr10:123922850-123923542 | chr10:126135809-126138896 | chr10:99446696-99447400 |
| chr11:18548060-18548629 | chr11:406491-407871 | chr11:45868742-45869413 | chr11:59383165-59383410 |
| chr11:64001572-64002712 | chr11:64072064-64073914 | chr11:65685100-65685364 | chr11:829284-833558 |
| chr11:842293-843396 | chr12:105724272-105725052 | chr12:11802752-11802983 | chr12:12509911-12510428 |
| chr12:131356094-131357228 | chr12:133337981-133338989 | chr12:14927291-14928023 | chr12:53441384-53441706 |
| chr12:57635240-57635572 | chr12:65563195-65564396 | chr13:25861234-25861794 | chr13:33161192-33161835 |
| chr13:37574792-37575223 | chr14:105218366-105218620 | chr14:24641053-24642220 | chr14:35098694-35099478 |
| chr15:42066488-42067230 | chr15:69745049-69745746 | chr16:30006578-30007874 | chr16:3332472-3333847 |
| chr16:69458264-69458626 | chr17:1394439-1394813 | chr17:30770960-30772137 | chr17:34890466-34891480 |
| chr17:42092143-42092432 | chr17:49337102-49338583 | chr17:55962573-55963229 | chr17:7832532-7833164 |
| chr19:1103722-1105758 | chr19:12551471-12551877 | chr19:1969438-1970136 | chr19:35760457-35760865 |
| chr19:46288988-46289934 | chr19:46366202-46366605 | chr19:50059574-50060175 | chr19:50379396-50381565 |
| chr20:31070189-31072676 | chr20:36148603-36150136 | chr21:38362015-38362868 | chr22:24384134-24384405 |
| chr22:40766459-40767033 | chr22:45559298-45560172 |  |  |

**Table 24.** Methylation sites associated with AZ628 drug sensitivity were located in 91 known transcription factor binding regions.

| ARID3A | ATF1 | ATF2 | BACH1 | BRCA1 | CBX8 | CEBPB | CEBPD |
| --- | --- | --- | --- | --- | --- | --- | --- |
| CHD4 | CREB1 | CREBBP | CTCF | CUX1 | E2F1 | E2F4 | E2F6 |
| EGR1 | ELF1 | ELK1 | ELK4 | EP300 | ESRRA | ETS1 | EZH2 |
| FOS | FOXA1 | FOXA2 | FOXM1 | FOXP2 | GABPA | GATA1 | GATA2 |
| GATA3 | HCFC1 | HDAC1 | HDAC2 | HNF4A | IKZF1 | IRF1 | IRF3 |
| JUN | JUND | KAT2B | MAX | MAZ | MTA3 | MXI1 | MYBL2 |
| MYC | NCOR1 | NFATC1 | NFIC | NFYA | NFYB | NR2C2 | NR2F2 |
| NRF1 | PAX5 | PML | PRDM1 | RELA | REST | RFX5 | RXRA |
| SIN3A | SMARCB1 | SP1 | SP2 | SP4 | SPI1 | SREBF1 | SREBF2 |
| SRF | STAT1 | STAT2 | STAT3 | STAT5A | SUZ12 | TAF1 | TAL1 |
| TBP | TCF12 | TCF7L2 | TEAD4 | TRIM28 | USF1 | USF2 | YY1 |
| ZBTB7A | ZEB1 | ZNF143 |  |  |  |  |  |

**Table 25.** Predicted methylation sites related to Dasatinib drug sensitivity, 98 of which were located in the transcription factor binding region.

| chr1:113217475-113218097 | chr1:11714217-11715304 | chr1:154973250-154974358 | chr1:180991719-180992149 |
| --- | --- | --- | --- |
| chr1:205781870-205783010 | chr1:21978101-21978764 | chr1:24306363-24307017 | chr1:247241579-247242201 |
| chr1:28052145-28053002 | chr1:94146321-94147504 | chr1:95699725-95700142 | chr2:11295559-11296006 |
| chr2:183730808-183731357 | chr2:190539026-190539533 | chr2:191878608-191879253 | chr2:230932468-230933550 |
| chr2:37383901-37384402 | chr3:172468372-172468845 | chr3:183959061-183959747 | chr3:184056419-184056671 |
| chr3:38206498-38207642 | chr3:50312687-50314419 | chr3:53032813-53033116 | chr4:146402870-146403983 |
| chr4:151500763-151501299 | chr4:73934944-73935650 | chr4:76861089-76862426 | chr5:151150014-151152086 |
| chr6:24357719-24358309 | chr6:25726454-25726722 | chr6:32811494-32811839 | chr6:32820849-32822370 |
| chr6:76311330-76312563 | chr7:1126578-1126910 | chr7:148395430-148397002 | chr7:2353629-2354075 |
| chr7:43965806-43966400 | chr7:5085411-5086342 | chr7:8473139-8475199 | chr7:95064073-95064409 |
| chr7:99724482-99725808 | chr8:104426894-104427823 | chr9:130742515-130743486 | chr9:139439041-139441532 |
| chr9:2717903-2718953 | chr10:103603166-103603642 | chr10:104473399-104474711 | chr10:120863620-120864216 |
| chr10:123922850-123923542 | chr10:128973590-128974064 | chr11:118781060-118781732 | chr11:118977878-118978785 |
| chr11:36531167-36531915 | chr11:45868742-45869413 | chr11:63993589-63993834 | chr11:64001572-64002712 |
| chr11:65685100-65685364 | chr11:842293-843396 | chr12:104697348-104697984 | chr12:105724272-105725052 |
| chr12:119772353-119772594 | chr12:133337981-133338989 | chr12:47629034-47629680 | chr12:50297580-50297988 |
| chr12:53441384-53441706 | chr13:37574792-37575223 | chr13:99228319-99229576 | chr14:21737406-21738094 |
| chr14:97263559-97264274 | chr16:3332472-3333847 | chr16:56671937-56672530 | chr16:58549027-58550016 |
| chr16:89487760-89488158 | chr17:1063942-1064304 | chr17:34890466-34891480 | chr17:42092143-42092432 |
| chr17:73149830-73150885 | chr17:7342829-7344028 | chr18:23713728-23713953 | chr18:46474931-46479364 |
| chr19:1103722-1105758 | chr19:12551471-12551877 | chr19:17877468-17877777 | chr19:18061195-18061473 |
| chr19:2474610-2476828 | chr19:41222598-41223162 | chr19:42579935-42580588 | chr19:46236428-46236957 |
| chr19:46366202-46366605 | chr19:50713630-50714073 | chr19:55591905-55593980 | chr19:632697-633678 |
| chr19:6425485-6425843 | chr20:30311065-30311872 | chr20:36148603-36150136 | chr21:38362015-38362868 |
| chr22:24384134-24384405 | chr22:40766459-40767033 |  |  |

**Table 26.** Methylation sites associated with Dasatinib drug sensitivity were located in 87 known transcription factor binding regions.

| ARID3A | ATF2 | BACH1 | BRCA1 | CBX8 | CEBPB | CEBPD | CHD4 |
| --- | --- | --- | --- | --- | --- | --- | --- |
| CREB1 | CREBBP | CTCF | CUX1 | E2F1 | E2F4 | E2F6 | EGR1 |
| ELF1 | ELK1 | ELK4 | EP300 | ESRRA | ETS1 | EZH2 | FOS |
| FOSL2 | FOXA2 | FOXM1 | GABPA | GATA1 | GATA2 | GATA3 | HCFC1 |
| HDAC1 | HDAC2 | IKZF1 | IRF1 | IRF3 | JUN | JUND | KAT2B |
| MAX | MAZ | MTA3 | MXI1 | MYBL2 | MYC | NCOR1 | NFATC1 |
| NFIC | NFYA | NFYB | NR2C2 | NR2F2 | NRF1 | PAX5 | PML |
| POU2F2 | RELA | REST | RFX5 | RXRA | SIN3A | SMARCB1 | SP1 |
| SP4 | SPI1 | SREBF1 | SREBF2 | SRF | STAT1 | STAT2 | STAT3 |
| STAT5A | SUZ12 | TAF1 | TAL1 | TBP | TCF12 | TCF7L2 | TEAD4 |
| TRIM28 | USF1 | USF2 | YY1 | ZBTB7A | ZEB1 | ZNF143 |  |
